# Perceptions of ecosystem services: Comparing socio-cultural and environmental influences

**DOI:** 10.1371/journal.pone.0276432

**Published:** 2022-10-27

**Authors:** Miriam Thiemann, Rebekka Riebl, Maria Haensel, Thomas M. Schmitt, Manuel J. Steinbauer, Theresa Landwehr, Ute Fricke, Sarah Redlich, Thomas Koellner

**Affiliations:** 1 Professorship of Ecological Services, Bayreuth Center of Ecology and Environmental Research (BayCEER), University of Bayreuth, Bayreuth, Germany; 2 Sports Ecology, Bayreuth Center of Ecology and Environmental Research (BayCEER) & Bayreuth Center of Sport Science (BaySpo), University of Bayreuth, Bayreuth, Germany; 3 Department of Animal Ecology and Tropical Biology, Julius-Maximilians-University, Würzburg, Germany; CSIR National Environmental Engineering Research Institute (CSIR-NEERI), INDIA

## Abstract

Ecosystem services such as food provisioning, climate regulation, nutrient cycling, or recreation in open landscapes underpin human wellbeing. They are highly dependent on land use, land cover and utilization pattern as well as environmental factors like climate, topography and soil. In consequence, ecosystem services supply shows a high spatial variability. However, it is less clear if the perception of the importance of ecosystem services is similarly heterogeneous in space and amongst societal actors. The aim of this large-scale study was to explore whether land cover and climate gradients as well as socio-cultural factors influence the perceptions of ecosystem services of four groups of societal actors: citizens, farmers, foresters and nature managers. Spatially explicit survey data of 3018 respondents allowed to gain insight into the distribution of perceived importance of 21 ecosystem services in the federal state of Bavaria, Germany together with the respondents’ socio-cultural characterisation (e.g. gender, education and hobbies in nature). Responses were analysed through descriptive statistics, redundancy analysis, and Generalized Linear Models. Results reveal that the perceived importance of many ecosystem services was consistently high across groups, although perception differed for some ecosystem services (e.g. production of energy plants and timber as well as recreation in urban green space). Compared to other actor groups, farmers attributed slightly lower importance to all ES except provisioning services. Socio-cultural factors better explained variability in perceived importance of ecosystem services than land cover and climate gradients. This might be either explained by the fact that the environmental gradients vary not strong enough in our case study or that they do not shape the perceptions of respondents. A limitation of the study is that the sample of respondents obtained is not representative for the population, but biased towards persons interested in the topics of the survey. Still the consensus indicated by the overall positive perception of ecosystem services among respondents highlights the integrative potential of ecosystem services when included in decision-making.

## 1. Introduction

Land use and spatial planning decisions entail trade-offs between different goals that respectively benefit or harm aspects of human well-being. Ecosystem services (ES) research can contribute to revealing those trade-offs. Next to management aspects, natural conditions drive the high spatial variability of ES like food provisioning, climate regulation, nutrient cycling, or recreation in open landscapes [1]. Valuation of ES supports policymakers in assessing consequences from different management options [2]. The contribution of ES to human well-being, made transparent through ES valuation, can be expressed monetarily, biophysically or by gathering data about how people perceive and evaluate ES in their region [3], the socio-cultural valuation.

Socio-cultural valuation is a form of depicting the social demand for ES. Generally, ES research is said to have a bias towards biophysical and monetary values [4]. In socio-cultural valuation, values are understood differently than in monetary or biophysical valuation [5]. It refers to a psychological concept where so-called held values are “principles or ideas that are important to people, such as notions of liberty, justice or responsibility” [6]. Perceptions of ES as a concept within socio-cultural valuation can loosely be defined as a way of observation, understanding, interpretation and evaluation [7]. Antrop [8] stated that “perception, as complex learning processes, analyses the observation immediately and interactively and links the results with our knowledge and past experience.” Derkzen et al. [9] highlight that including socio-cultural valuation is useful to assess people’s preferences complementing monetary approaches, e.g., willingness to pay, because it is independent of respondents’ socio-economic situation. Socio-cultural valuation is considered to be particularly suitable in revealing intangible values [10]. While for biodiversity conservation large-scale surveys regarding people’s values and perceptions are already implemented and feed into respective policies [11], for ES such comprehensive data are still lacking. Thus, it is relevant to investigate individuals’ valuation of ES within different social contexts [12, 13].

Several studies explored different sets of factors influencing the perception of ecosystem services. Firstly, socio-cultural information of the respondents was commonly gathered in previous studies about ES perception. Typically, gender [14], age [14, 15], education level [15, 16] and length of residency [17] were considered. Knowledge about concepts like biodiversity, soil fertility and the region-specific conditions shaped farmers’ perception of agricultural management effects on ES in a study by Lamarque et al. [18]. Secondly, perception or attributed importance of ES also varied between different societal actor groups [13–15, 19]. Some studies distinguished between societal actors having a direct influence on land cover and land-use and the public with a solely indirect influence [20–22]. Commonly, sampled societal actor groups were farmers, environmental experts, tourists or residents. Lastly, gradients of land use and cover as well as climate were investigated as influencing factors. This is because the perception of people and the importance they attribute to ES can vary depending on their place of residence and the ecosystem they are surrounded by. For example, different sets of cultural ecosystem services were perceived as important along an urban to peri-urban gradient in Berlin [23, 24]. In studies along a rural to urban gradient, rural population valued provisioning services higher than the urban population which instead rated regulating services [14] or cultural services [17] higher. The climate gradient may play a role in ES perception since experiences of people with their environment have been shown to be an influential factor. Frondel et al. [25] found that personal experience with adverse events like storms, floods and heat waves increases the perceived risk of climate change, especially when personal damage occurred. This suggests that ES perception may also change along a climate gradient represented either by extremes or by steadily changing mean temperature or precipitation. Though literature already provides good estimates on influencing factors of ES perceptions, most surveys on perception of ecosystem services cover only either the influence of environmental gradients or socio-cultural factors. Hence, there is a lack of comprehensive research investigating the influence of multiple sets of factors on ES perceptions in one large-scale study.

In this study, we investigate the perceived importance of ES by different societal actors indicating their held values. This was done on a large scale covering representative regions of Bavaria, Germany. For this purpose, a survey among four societal actor groups was conducted. Perceptions of farmers, foresters and nature managers, who have the power to influence land use directly, are expected to differ from the group of citizens with indirect influence only. In addition, differences between directly influencing societal actors are expected due to their different professional backgrounds. The data collection was designed to cover gradients of temperature, precipitation and different land covers to assess the impact of these environmental factors on ES perception. For instance, we expected that respondents from Southern Bavaria, where regions experience higher precipitation, show less awareness for services like climate regulation and groundwater formation than respondents from the drier regions of North-Western Bavaria. The land cover gradient can be expected to play a role, since different land cover environments provide different types of ES, increasing or decreasing the supply of certain ES.

We hypothesize that the perceived importance of ES varies depending on the individual’s socio-cultural background and the environment they live in. Specifically, we expect differences to occur between the four societal actor groups surveyed in this study and along the implemented climate and land cover gradients.

## 2. Methods

### 2.1 Study region

This study was conducted in Bavaria, Germany, populated by roughly 13 million inhabitants [26]. Land cover of the study region (70.500 km^2^) is dominated by agriculture (47%), followed by forest (35%), settlement and transport (12%) and other land cover such as vegetation-free, peatland, swamp and water areas (6%) [27]. These land cover types vary across Bavarian regions with, for example, higher shares of settlement and transport in the urban areas around the cities of Munich and Nuremberg.

The mean annual air temperature is 7.8 °C with relatively strong differences between regions, Lower Franconia has a mean annual air temperature of 10 °C, the Allgäu region including parts of the Alps 6 °C [28]. Global warming is also causing a warming trend in Bavaria. Between 1881 and 2014, the mean annual temperature increased by 1.4 °C [28]. Nine out of ten of the warmest years recorded occurred within the last 20 years [29]. The mean annual precipitation in Bavaria is 945 mm with a North-South gradient covering lower annual values between 600 and 700 mm in middle and North-western Bavaria and peak values of 1800 mm in the Alpine regions in the South [28].

### 2.2 Survey implementation

**Fig 1:**
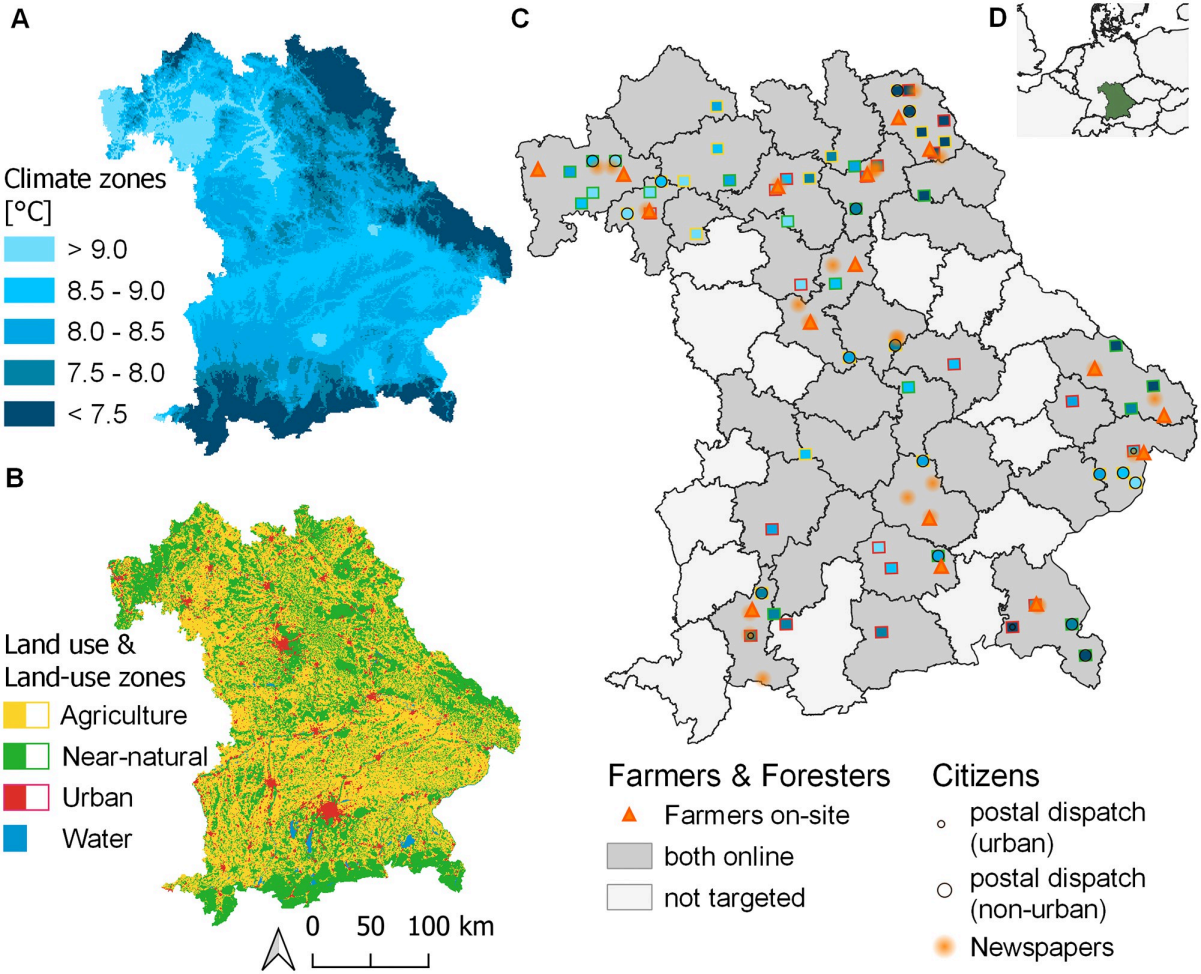
Study region and sampling design: A) Climate zones based on mean annual air temperature (reference period 1981–2010, DWD), B) land-use zones are based on CORINE Land Cover 2012 C) Sampling gradient shown for the 60 focal quadrants with filled colour from A) and border colour from B). Locations of surveys are shown for farmers (orange triangles: on-site locations of Offices of Food, Agriculture and Forestry; grey regions: online distribution via Offices and Agricultural Associations), foresters (online distribution via Offices and Forestry Associations to private and corporate foresters; 20 state foresters per climate zone were addressed via the Bavarian State Forestry BaySF, not depicted), citizens (black circles, urban quadrants with r = 1.5 km, non-urban quadrants r = 3 km to account for different population densities; orange hatches: location of editorial offices of newspapers that invited readers to participate in the survey via articles, r = 10 km is not true to scale of the newspapers’ range of influence); nature managers were contacted independent of their location within the study region and are thus not shown in C. D) Location of the study region Bavaria (green), in Germany and Central Europe.

In our online survey, we addressed four different societal actors: citizens, farmers, forester and nature managers (professionals and volunteers managing near-natural areas). Our sampling strategy is based on Redlich et al. [30] who identified 60 quadrants (5.8 km x 5.8 km) across Bavaria that covered a representative gradient of land cover and climate (see Fig 1A and 1B). Quadrants counted as ‘agriculture’ entail more than 40% arable land and managed grassland, ‘near-natural’ more than 85% near-natural vegetation with a minimum of 50% forest, ‘urban’ more than 14% housing, industry and traffic infrastructure, respectively. Climate zones of the quadrants were assigned using the Climatological Standard Normal of 1981–2010, whereas for the analysis, the period 1990–2019 was used to include recent years more relevant to participants’ perceptions.

The survey was conducted from January to July 2020. The sampling approaches were specific to the respective group of societal actors (Fig 1C). For the group of citizens, 44,244 households received a postal invitation (S1 Fig in S1 File) with a link to the online survey. The households were chosen in accordance with the study design in different climatic and land cover areas. Per quadrant, each household in postal delivery districts within a specific radius (r = 1.5 km for urban areas, r = 3 km for agricultural and near-natural areas) around the quadrant centroid was contacted. The radius was set depending on the land-cover type of the quadrant in a way to achieve equal amounts of invitations sent out as much as possible per quadrant type. Additionally, local newspapers in the quadrants were contacted and invited to report about our research and to provide the link to the online survey (see S1 Table in S1 File for a list of postal delivery districts and successfully contacted newspapers).

The proportion of land managers (farmers, foresters, nature managers) is low compared to the general Bavarian public. Therefore, these three societal groups were contacted in a more targeted approach. The group of farmers was surveyed via cluster sampling. In a first survey phase, farmers applying for subsidies for agri-environment-climate measures (a majority of farmers participate in those measures) at local agricultural offices were invited to participate in the survey. The chosen 12 out of 47 Bavarian Offices of Food, Agriculture, and Forestry (Ämter für Ernährung, Landwirtschaft und Forsten ÄELF) covered the gradient of the study quadrants. (see S2 Table in S1 File for a list of the local offices). Members of the research team acted as facilitators in the agency and invited farmers frequenting the respective office to fill out the survey on tablets while queuing in the waiting room. This was a unique opportunity to survey this actor group as data protection regulations restrict the access to farmers’ addresses to send out questionnaires. The second part (phase of general subsidy application) of the planned in person sampling period had to be cancelled due to the Covid-19 pandemic. As a replacement, local agencies of the Bavarian Association of Farmers (Bayerischer Bauernverband) sent a link to the online survey to their members via e-mail.

The group of foresters included trained foresters, forest managers, and forest owners of state, private and corporate forests in Bavaria. This group received an e-mail invitation to the online survey via the same offices in which farmers were approached as well as via the Bavarian State Forestry (Bayerische Staatsforsten BaySF). In the latter case, twenty forest districts per climate zone (in total 100 districts) were selected and via the central BaySF office e-mails with the link to the survey were sent to the district foresters. In consequence, the respondents have similar educational background and might be influenced by the organisations’ official policy of nature protection and climate change.

As nature managers, we considered all people working in any kind of job or volunteering function that involves managing near-natural areas and urban green spaces. Examples are nature conservation authorities, municipal administrative divisions of urban green area management and landscape architects. Those agencies were contacted and invited to spread the link to the online survey Bavaria-wide via e-mail.

For all groups, surveys were conducted anonymously and analysed separately from any personal contact data, which could be optionally provided. To comply with protection of data privacy, the purpose of the survey and that data will be used for scientific publications and to inform policy makers was stated at the beginning of the questionnaire. At its end, participants actively agreed that their answers can be stored and scientifically analysed. Due to this consent given by the respondents to take part in the research and no data on health was requested, we did not seek approval of the institutional ethics committee to conduct this study.

### 2.3 Survey content

The use of structured questionnaires is a common method in socio-cultural valuation of ES [31]. The questionnaire used in this study was tailored towards perception of ecosystem services and climate change in Bavaria. It was structured in following sections Q1: introduction, Q2: personal relationship to nature and landscape, Q3: perceived importance of ES, Q4: knowledge on ES, Q5: opinion on land use trade-offs, Q6: spatial preferences and values of ES, Q7: opinions on enhancement of ES, Q8: perception of climate change in general, Q9: perception of climate change on land owned/managed by respondent, Q10: climate change adaptation, Q11a: management practises (specific for foresters, farmers and nature managers), Q11b: socio-cultural data, Q12: follow-ups and feedback. The questionnaire was implemented in the online software Qualtrics. An export of the questionnaire for citizens is shown exemplarily in supporting information B. Some questions were tailored towards the specific societal actor groups (e.g. management practices of farmers), but only answers from consistently used questions were used in this study. Our study focused on the sections Q1: introduction, Q2: relationship to nature and landscape, Q3: perceived importance of ES, Q4: knowledge on ES, Q11b: socio-cultural data, and Q12: follow-ups & feedback. Respective questions are listed in S3 Table in S1 File. We displayed a total of 21 ES falling into four broad categories (S4 Table in S1 File) and provided some examples for each ES (S5 Table in S1 File). The basis for the selected ES and their classification were the TEEB categories [32] and the ES classification in Rabe et al. [33]. However, not the whole list of 21 ES was displayed to each respondent to shorten the individual survey. Instead, one of four subsets with seven ES was randomly assigned to respondents (S6 Table in S1 File). Three subsets contained fixed sets of ES and the fourth subset contained seven ES chosen randomly from the pool of 21 services. Within the subsets, the order of displayed ES rotated to avoid framing or distorting effects. Respondents were asked to attribute the importance they perceive for each ES in Bavaria. The scale for this question was a five-point Likert-type scale ranging from “very unimportant” to “very important” [34]. Respondents could also choose “I do not know” as an answer. However, this only occurred in 1% of cases and was thus excluded in the analysis. Throughout the questionnaire, the term “services of nature and landscapes”, which is also the legal term in most of the German nature protection law [35], was used instead of ES to make the questions easily understandable, even for respondents being not familiar with the concept of ES.

### 2.4 Respondent characterization

**Fig 2:**
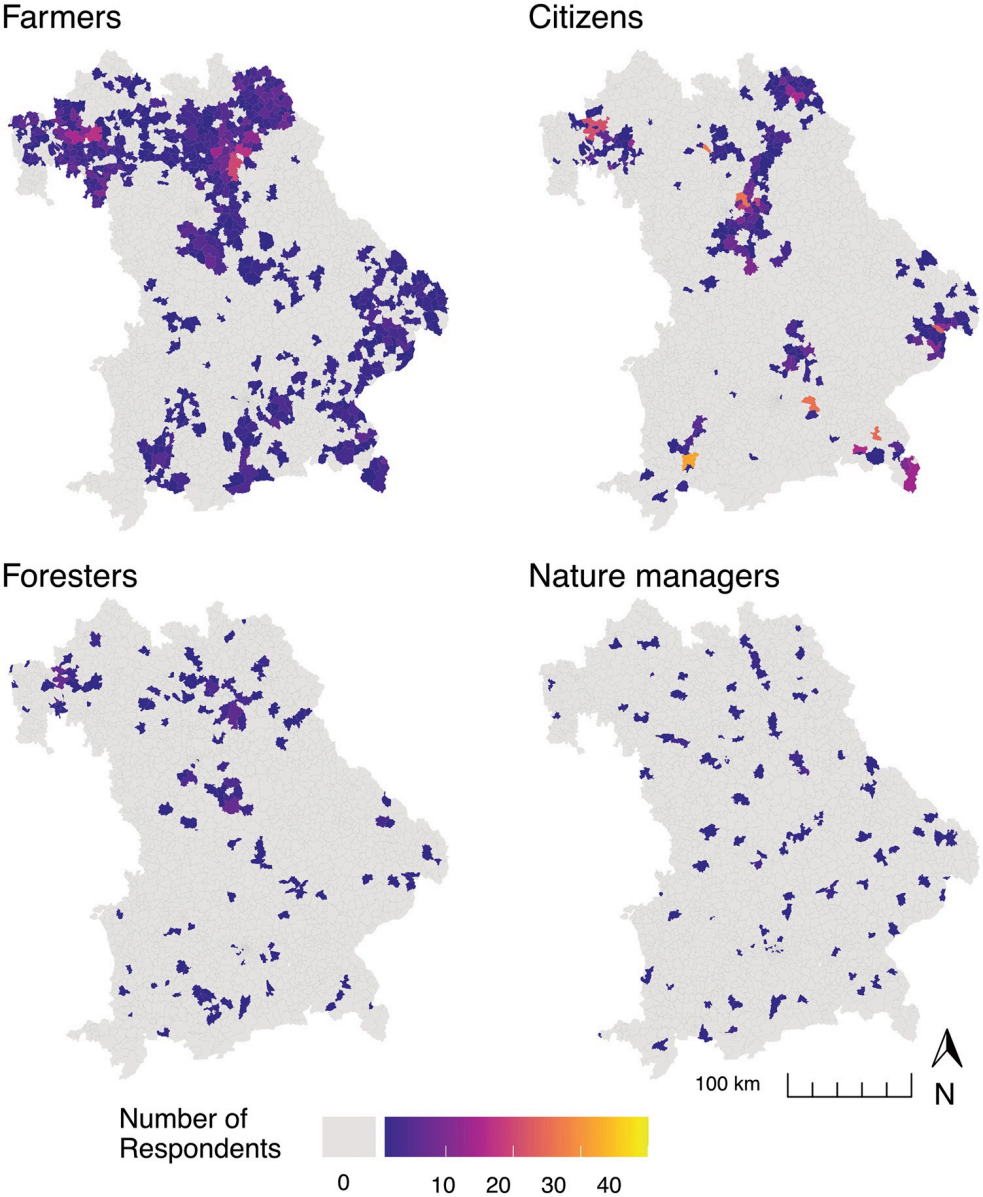
Residence of respondents that answered the question regarding their perceived importance of ES (n = 2343, as 675 respondents did not provide their postal code), differentiated by societal actor group. Coloured polygons represent the postal code areas.

A total of 3295 persons participated in the survey. In this study, we included all respondents answering the question regarding their perceived importance of ES (n = 3018). The majority belonged to the group of farmers (n = 1676), followed by citizens (n = 948), foresters (n = 225) and nature managers (n = 169). The spatial distribution varied amongst participants (Fig 2). Compared to farmers and citizens, the residence of nature managers and foresters is more dispersed across Bavaria. This is due to the circumstance that these groups are only represented in low numbers in Bavaria and, unlike farmers, could not be targeted at a specific office of agriculture. Across all groups, 675 respondents (22%) did not indicate their postal code and could thus not be considered in the spatially explicit analysis parts. The participation along the climate and land use gradients is uneven (S2 Fig in S1 File).

Less than half of the participants (n = 1313) took less than or exactly 20 minutes (which was the duration announced in the introduction) to fill out the questionnaire (S3 Fig in S1 File). A relatively high share of participants (total n = 1233) provided personal contact information to receive a short report on the survey results, to participate in future surveys or in citizen science projects. This reflects their generally high interest in the topic of the survey. In addition, about one third of respondents across groups indicated that they know the term ES.

The distribution of socio-cultural features in the sample differs between actors (S7 Table in S1 File). The most common type of school attended by survey participants is higher secondary school. However, a strong educational difference exists between the groups. For instance, surveyed foresters typically hold a degree from higher secondary school (81%), whereas only one third of surveyed farmers frequented this school type (32%).

### 2.5 Data analysis

Data analysis was implemented in the statistical software R version 4.0.3. Land cover data was prepared with QGIS version 3.14.16. Firstly, we provide an overview of the results via descriptive statistics, creating Likert plots of respondents’ answers on each ES based on all four subsets, using the *HH* package in R Studio [36]. For each ES an index of disagreement was calculated as the squared distances between the weighted sum of Likert classes of the answers of one actor group compared to all actor groups. Then, the mean for all actors was calculated and reported.

Secondly, as an exploratory step, we conducted a redundancy analysis (RDA) with the *vegan* package in R [37]. This technique can be used for exploratory analysis of relationship between variables and has been applied in other studies examining factors influencing ES perceptions [14, 15]. As respondents only replied to subsets of seven out of the 21 ES (see 2.3), each respondent has missing values for the remaining 14 ES that were not displayed to them in the survey. We correspondently carried out a separate RDA for each of the three fixed subsets of ES (S6 Table in S1 File) to ensure complete cases of dependent and explanatory variables.

Thirdly, a correlation plot depicting significant correlations based on the Spearman correlation coefficient is drawn for the relationship of all ES with the land cover and climate variables.

Fourthly, generalized linear models (GLM) were implemented for each of the ES to identify how response behaviour is influenced by explanatory variables, especially the gradient variables (variables listed in Table 1). GLMs were implemented with Poisson family error. Forward stepwise selection was used to only include relevant variables in the final model. This approach is based on the Akaike’s Information Criterion (AIC) [38].

**Table 1:** Explanatory and response variables used in the analysis.

	Characteristics	Categories	Source
Response variables	Perceived importance of 21 ecosystem services	Very unimportant (1) Unimportant (2) Indifferent (3) Important (4) Very important (5) I don’t know (excluded)	Survey
Explanatory variables	Societal actor	Citizen, farmer, forester, nature manager	Survey design
	Gender	Male, female, diverse, no answer	Survey
	Age	<18, 18–25, 26–30, 31–35, 36–40, 41–45, 46–50, 51–55, 56–60, 61–65, 65–70, >70	Survey
	Number of hobbies in nature	*List of five suggested activities and free text field for other activities (Total of six activities possible)*	Survey
	School Education	Lower secondary school Middle secondary school Higher secondary school	Survey
	Knowledge of ES term	Yes, No	Survey
	Postal code	< *free* >	Survey
	Climate variables	Annual mean temperature in °C, Precipitation in mm/year	DWD, based on 1990–2019 period
	Land cover variables	Urban areas in %, Agricultural areas in % and Near-natural areas in % of postal code area	CORINE Land Cover data 2018

Socio-cultural explanatory variables are based on the survey, whereas environmental variables are based on external sources. The CORINE Land Cover 2018 data [39] was the basis for the land cover gradients (see Fig 1). For the analysis, ‘urban’, ‘agricultural’ and ‘near-natural’ were used as main categories summarizing the 44 original categories (S8 Table in S1 File). Climate data was provided by the German Weather Service [40]. The mean annual air temperature and mean precipitation were calculated based on the period 1990–2019. The land cover and climate information were calculated based on the available scale of the survey, the postal code areas. Then, land cover and climate variables were assigned to each respondent based on the indicated postal code of their residence. The individual distribution of each gradient variable across Bavaria is shown in S4 Fig in S1 File.

The response variables “importance of the respective ES” were shown between “very unimportant (- -) and “very important (+ +)” in the questionnaire. In the analysis, we treated them as numeric variables (1–5) to enable a quantitative analysis, as it was also done by Wardropper et al. [19], for example. Later we discuss the implications of this transformation (section 4.3).

## 3. Results

### 3.1 Perceived importance of ecosystem services

**Fig 3:**
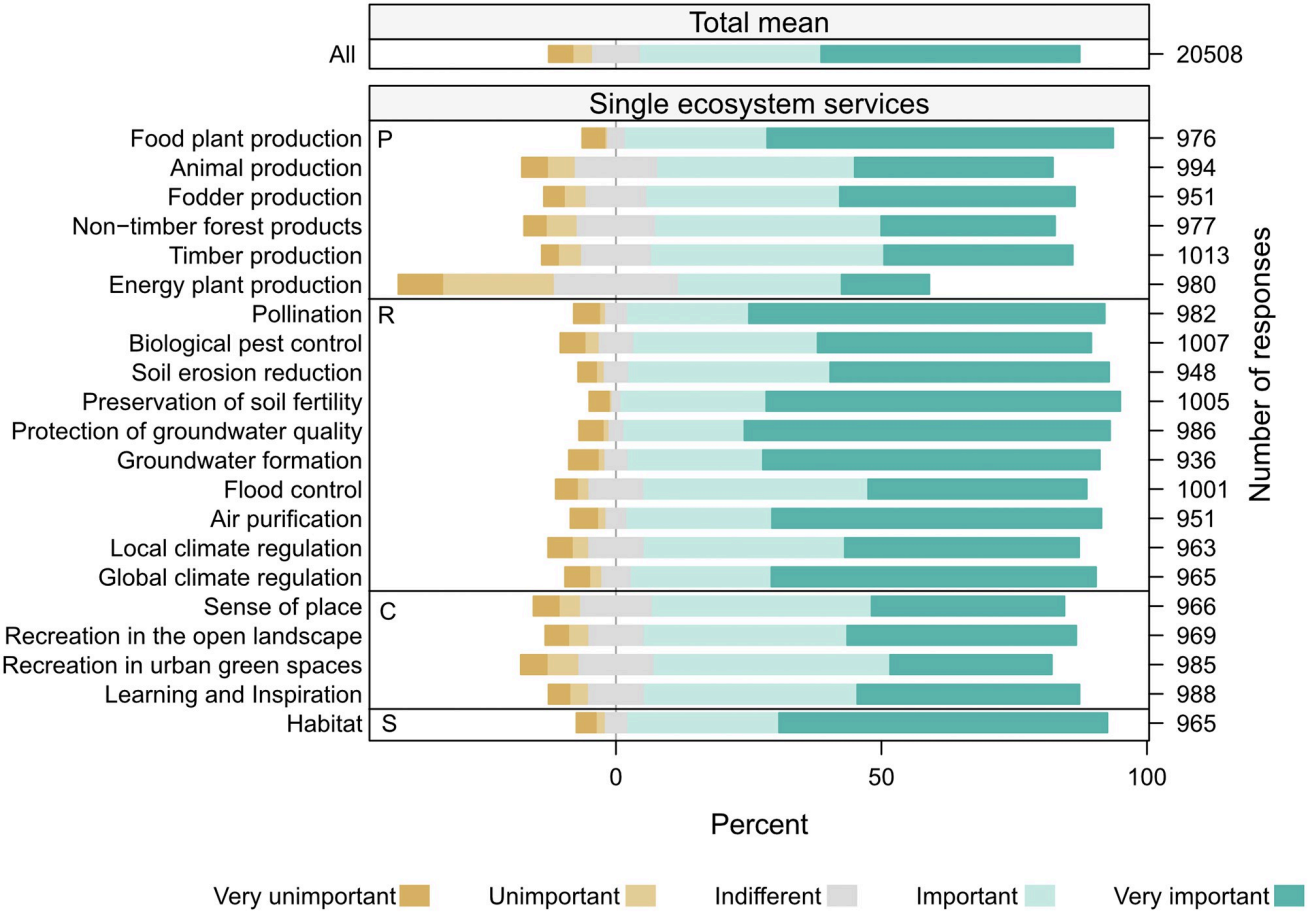
Overall perceived importance per ecosystem service ES (P: provisioning services, R: regulating services, C: cultural services and S: supporting services). The respective question was “How important are the following services of landscape and nature?” Segments in turquoise stand for the percentage share of answers in the important or very important category. Indifferent answers are split equally around zero percent. Segments in brown represent the percentage share of answers in the unimportant or very unimportant categories. Numbers on the right side of each row represent the number of responses for each respective ecosystem service. The high number of responses (i.e. 20508), is due to aggregated ES answers; each of the 3,018 respondents could give an answer for seven ES.

Overall, all ecosystem services were highly valuated as they were mostly rated as very important or important (Fig 3). Among the four ES categories, the habitat category received the highest attributed importance with a mean value of 4.43. The group of regulating services was ranked on average at 4.36, followed by provisioning services at 4.10 and cultural services at 4.00. Regarding single ES, the highest average values were given to preservation of soil fertility and protection of groundwater quality with 4.53 and 4.50, respectively. The service of energy plant production was the one service that scored comparatively low with a mean of 3.27.

**Fig 4:**
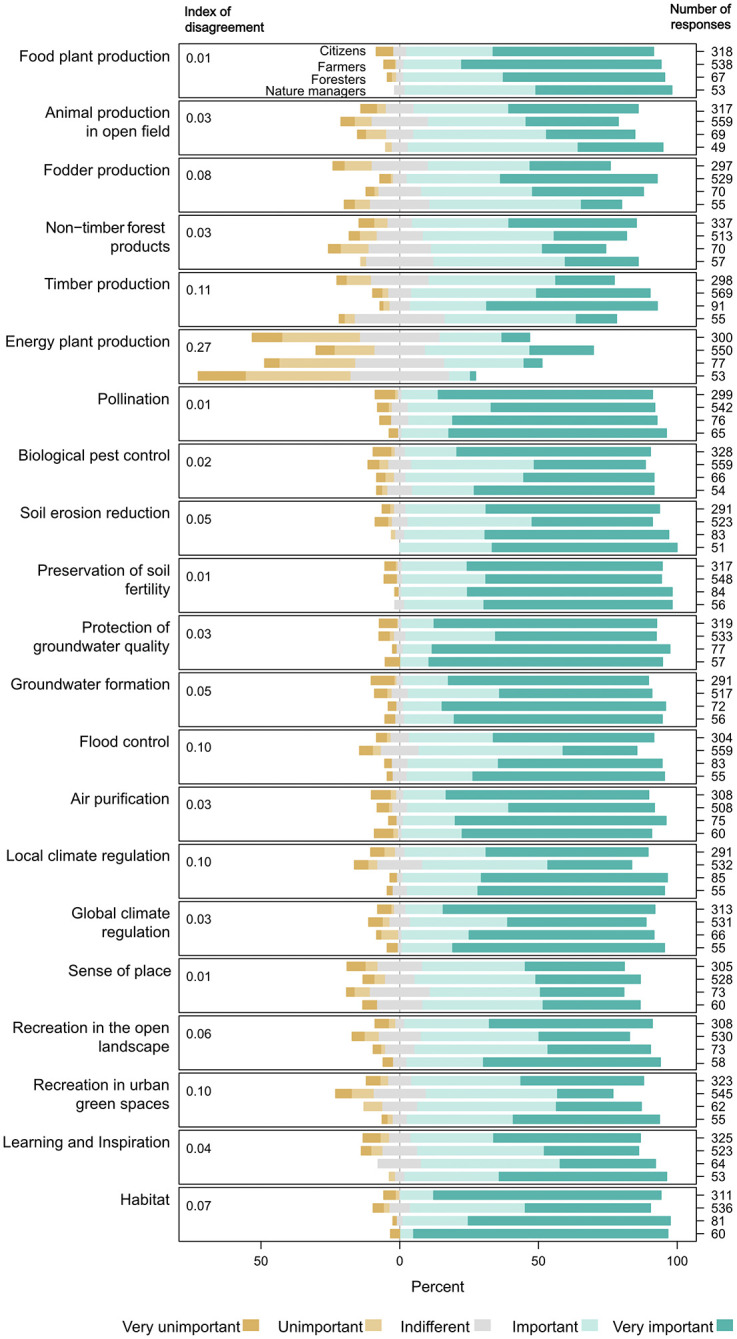
Perceived importance per ecosystem service and societal actor (i.e., citizens, farmers, foresters, and nature managers). The index of disagreement shows differences between societal actors in perceived importance of ecosystem services.

Comparing the four groups of societal actors reveals both similarities as well as differences regarding the perceived importance of ES (Fig 4). Citizens, nature managers and foresters often stated a higher importance for several ecosystem services compared to farmers. This was specifically the case for regulating, cultural and supporting services. Provisioning services were partly perceived as being more important by farmers and foresters. The highest disagreement between the actor groups was shown for energy plant production (0.27), followed by timber production (0.11), local climate regulation (0.10) and recreation in urban green space (0.10). High agreement with an index value of 0.01 each was achieved for food plant production, pollination, soil fertility and sense of place.

Our results suggest that the professional background of the societal actors influences their perception of ES that are related to their occupation (Fig 4). Foresters perceived timber production as more important than the other societal actors; farmers attributed a higher importance to energy plant production, food plant production, and fodder production than the rest. For nature managers, the habitat service was perceived as very important by over 90% of the group. Surprisingly, however, animal production in the open field received the lowest importance by farmers. For the regulating services, answers showed little variation between the societal actors. One slight distinguishment is visible in the share of the category “very important” ES. Farmers attributed it less often to regulating services than the other groups. Differences of perceived importance of ES depended on gender, school degree, knowing the term ES and number of outdoor activities, respectively (S9 Table and S5 to S8 Figs in S1 File). Most interesting was the increasing perceived importance of ES with rising number of outdoor activities as stated by respondents.

### 3.2 Exploratory analysis for explaining the perceived importance of ecosystem services

The basic pattern of the relationship between response and exploratory variables was revealed through a RDA for the three constant subsets of ES each. The explained variance of the RDAs is low, covering only up to ten percent in the first two axes (S9 Fig in S1 File). In the negative scores of the first axis of ES subset 1, only production of food plants has a low score and is more closely related to farmers and nature managers as well as those respondents from a region with a high share of agricultural land cover than the other ES. For subset 2, farmers are associated with the ES fodder production in permanent grassland. Air purification, pollination, groundwater formation, and protection of groundwater quality are related to those respondents who practice outdoor hobbies, who live in regions with high precipitation, and are close to a near-natural environment. For subset 3, the RDA reveals an association between energy plant production and timber production, which are related to farmers and respondents from regions with agricultural land cover. These are opposed to regulating and supporting ES that are associated with respondents with a high education level, a high number of hobbies in nature, and older participants.

Overall, most striking is that farmers are distinct from the rest of the groups in all three RDAs. The farmers perceive especially provisioning services as important but are located further away from regulating and cultural services in the biplots. Unawareness of the concept ES mostly points in the opposite direction of regulating and cultural services.

**Fig 5:**
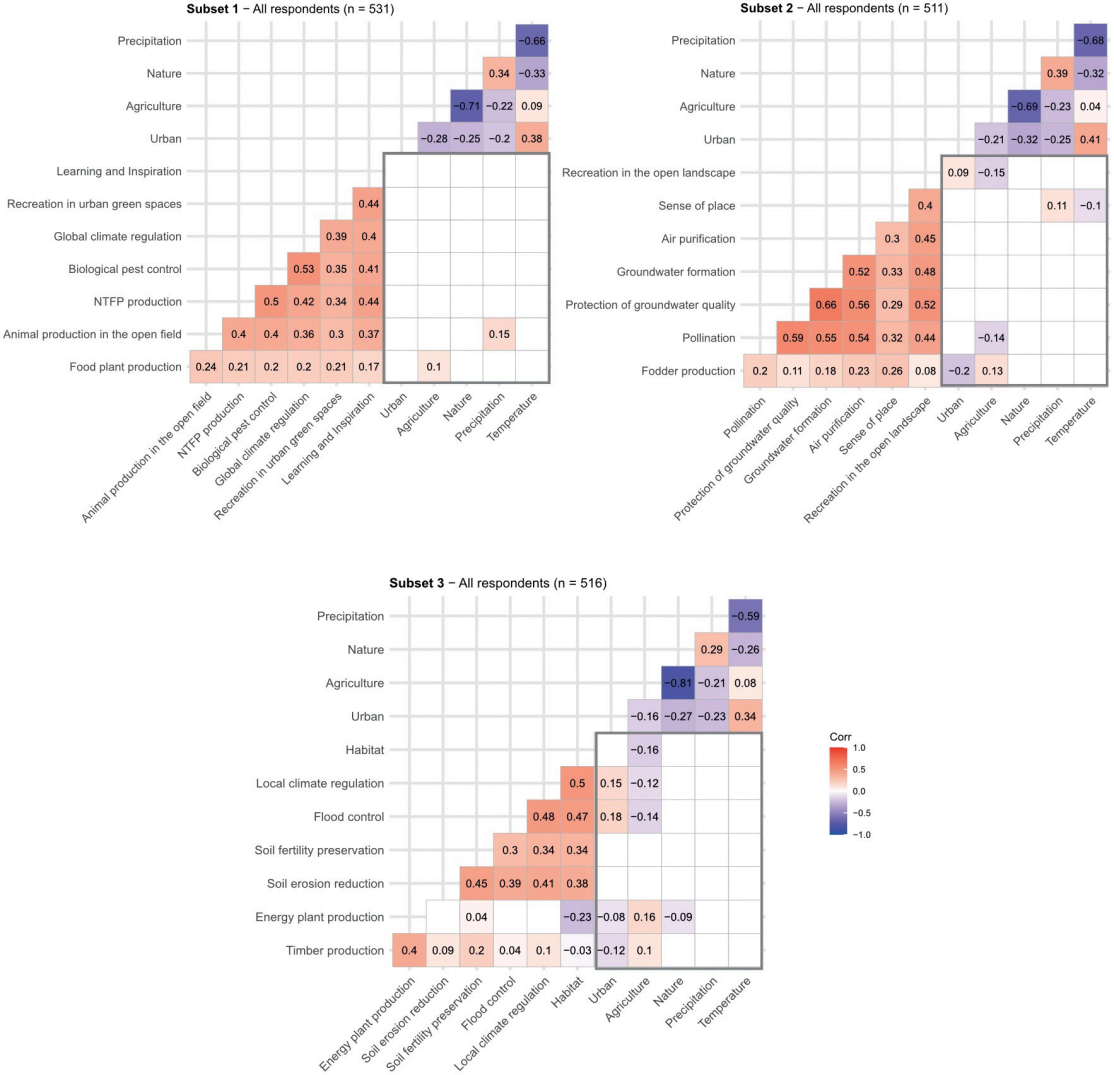
Correlation plots between ecosystem services and gradient variables. The grey rectangle frames the area of potential correlation between ecosystem services perceptions and gradient variables. Spearman correlation is calculated. Only significant correlations at p < 0.05 are shown, insignificant relationships are left blank.

Correlations among climate and land cover variables were generally low, except for precipitation and temperature, as well as agriculture and nature (Fig 5). All ES perceptions were positively correlated with each other, apart from energy plant production which was weakly negatively correlated with the perception of the habitat service. The plots show that positive and negative correlations exist between some of the ES and gradient variables. The two strongest correlations are negative correlations between the ES of recreation in open landscapes and agricultural land cover share (r_s_ = -0,15; subset 1) as well as between habitat ES and agricultural land cover share (r_s_ = -0.15; subset 3) and positive correlations between agricultural land cover share and energy plant production (r_s_ = +0,16; subset 3) as well as flood control and urban land cover share (r_s_ = +0.16; subset 3). However, as these correlations are relatively weak, our hypotheses that climate and land cover gradients influence ES perceptions cannot be corroborated through these results.

This relatively uniform spatial distribution is also supported by maps of the spatial distribution of the perceptions for each ES (S10 to S30 Figs in S1 File). The maps display the mean answers on postal code area level and give a broad impression about the spatial distribution of respondents and their perceptions of ES.

### 3.3 Multivariate analysis: Results of the regression analysis

The GLM for each ES as a dependent variable reveals slight differences in perceived importance. The coefficients are to be interpreted in the unit of the answer (ranging from “very unimportant” to “very important”, translated to 1 to 5 for the analysis). The coefficients are shown in Table 2. Each ES was analysed separately, thus all answers from all subsets were considered. The results are to be interpreted in comparison to the group of citizens as reference level. Regarding the hypothesis that differences exist between societal actors, the GLM confirms that being a farmer is often a significant impact factor for the attributed importance to ES. This is to be interpreted as a difference in comparison to the group of citizens as reference level. Farmers have a higher perceived importance of fodder production, timber production, and energy plant production than citizens. In addition, their perceived importance of flood control, local climate regulation, recreation in urban areas, and habitat is lower than for citizens. Foresters have a slightly higher perceived importance of timber production compared to citizens. Nature managers attribute the importance of energy plant production to be 0.23 units lower than citizens do. Practicing one additional outdoor hobby leads to an increase in perceived importance of recreation in open landscapes by 0.037. In that sense, a person with no outdoor hobbies compared to a person with six, the maximum possible in the survey, would perceive recreation in open landscapes by 0.222 less important.

**Table 2:** Coefficients from the Generalized Linear Models. The coefficients for the group of farmers, foresters, and nature managers are to be interpreted relative to the group of citizens. The reference level for middle secondary school is the higher secondary school category. Knowledge on ES was never significant and is therefore not shown. Spatial distributions of residuals are shown in S31-S40 Figs in S1 File.

Ecosystem Service	n	Mean	Farmer	Forester	Nature manager	Female	Middle secondary school	Outdoor hobbies	Nature gradient	Precipitation gradient
Animal production in the open field	712	3.97				0.084*				0.00014*
Fodder production	666	4.13	0.152***							
Non-timber forest products	704	3.94				0.090*				
Timber production	730	4.04	0.121**	0.200**						
Energy plant production	699	3.27	0.180***		-0.230*				-0.002*	
Flood control	718	4.15	-0.093*							
Local climate regulation	689	4.14	-0.111**							
Recreation in open landscapes	689	4.12						0.037*		
Recreation in urban areas	708	3.9					-0.129*			
Habitat	706	4.43	-0.109**							

*** p < 0.001,

** p < 0.01,

* p < 0.05

The hypothesized influence of climate and land cover gradients turned mostly out to be not suitable for explaining differences in ES evaluation and were thus not selected for the final GLM by forward selection. Only the variables precipitation and nature have significant impact on one ES each (S41 Fig in S1 File). The perceived importance of animal production in the open field changes with precipitation. Per increase of 100 mm of annual precipitation, the answers rise by 0.014 units of the answer scale. The perceived importance of energy plants changes with natural land cover. Per increase of 1% in natural area in the respondent’s residence area the perceived importance of energy plants decreases by 0.002. For the gradients of urban and agricultural land cover, as well as the temperature gradient, no significant influence on any of the ES were found.

## 4. Discussion

### 4.1 Perceived importance of ecosystem services in the context of socio-cultural variables

One aim of this study was to depict how membership in societal actors groups and socio-cultural characteristics influence the perceptions of a wide range of ES. Results suggest that most of the ES are perceived as very important with high mean values for each ES ranking between 3.9 and 4.5 out of 5 (except for a lower rating of energy plant production at 3.3). Among the four societal actor groups, farmers were the group with the most distinguished ES perception. The RDA (S9 Fig in S1 File) displays farmers close to provisioning services but apart from the rest of ES, which is confirmed in the GLM. Farmers thus have a higher perceived importance of the production of fodder, timber, and energy plants, which are all provisioning services. This is in line with results from several other studies on ES perceptions [17, 18]. Farmers state lower importance, however, for regulating and cultural services, specifically flood control and climate regulation, as well as recreation in urban areas and habitat. This could be explained by the fact that farming as a profession entails a closer relationship with the above listed provisioning services and that those services provide for their livelihood [19]. In that sense it is surprising that farmers are not located closer to the service of animal production in the open field in the RDA and that the group comparison shows that farmers answer lowest for this service. This might be explained by the fact that in large parts of Bavaria cattle is predominantly held in stables throughout the year [41]. Thus, the group of farmers might perceive fodder production generally as more important than specifically animal production in the open field.

Several further influencing factors lead to differences in perceptions depending on the respective ES. The GLM only finds a positive significant influence for recreation in open landscapes. This indicates that practicing activities in nature increases the valuation individuals show for nature’s services. This outcome is similar to the finding of Faccioli et al. [13] who stated that people engaged in outdoor recreation display a higher environmental attitude. Unlike in other studies [15, 42], age and education do not result in a significant difference in ES perception.

Preservation of soil fertility, protection of groundwater quality, and the production of food plants are the three services with the highest attributed importance. They are also highly rated services within each group of societal actors. Water-related services were also among the highest valued services in other studies on socio-cultural valuation of ES [3, 19, 21]. An explanation for the high perceived importance of those three services across societal actors might be that they are fundamental for everybody’s well-being, irrespective of profession, gender, or personal preferences.

Energy plant production deserves special attention since it is the single most unimportantly perceived service. Energy plant production was also identified as the least important service by other studies on ES perceptions [19]. One reason for the negative attitude might be that the cultivation of energy plants for the individual does not seem as indispensable for human well-being as other services like food plant production or air purification, where the benefit provided might seem more direct. Another driver for the negative perception of energy plants can be that their production has been associated with pesticide contamination, the expansion of corn monocultures [43] and the ploughing up of grasslands [44].

### 4.2 Perceived importance of ecosystem services in the context of land cover and climate gradients

The hypothesis of this study was to find varying perceived importance of ES along a land cover and climate gradient. In previous studies, it was found that rural societies value provisioning services higher while urban areas put emphasis on regulating services [14, 45]. This difference was then explained for example by the suggestion that provisioning services are more tangible in rural areas [46]. Applied to the present survey, such a gradient should have translated into the land cover variables having an influence on the perception of certain ES. Also, it would have seemed plausible that the temperature variable affects the perception of local or even global climate regulation, for example. However, such a pattern was not observed, with two exceptions.

Firstly, precipitation has a positive impact on animal production in the open field only. The full written version of this ES in the questionnaire was: “Animal production in the open field (e.g. milk cows and ox fattening on pastures)”. When looking for a reason for this, it can be examined that within Bavaria, especially the Southern regions near the Alps experience high annual precipitation. There, in turn, animal production on pastures is widespread. In Upper Bavaria and Swabia, the two Southern regions in Bavaria bordering the Alps, almost 50% of the agriculturally used land area is occupied by permanent grasslands. In the rest of Bavaria the same share ranges between 20% and 30% [47]. The view of cows on meadows is perceived as part of the traditional cultural scenery. It is surprising, however, that the same effect was not found for fodder production. Secondly, natural land cover in the vicinity has a negative influence on the perception of energy plant production. This could be driven by people living in areas with a high share of near-natural land cover appreciating their natural surroundings. Also, a high share of natural land cover naturally means less agricultural land cover. Thus, respondents from these near-natural areas are less likely a farmer and farmers respond higher for energy crop production.

Agricultural land cover in respondents’ residence areas seemingly has some influence on ES perception. It is visually clearly distinguished in the RDA biplot but not found as a significant variable in the GLM. This might be the case since many of the farmers can be expected to live in agricultural areas. Thus, the position of agricultural land cover in the RDA can be driven by respondents being a farmer.

When attempting to understand why the implemented land cover and climate gradients did not have the hypothesized influence, several reasons come to mind. Firstly, two respondents with the same amount of annual precipitation can live in two locations far apart from each other. However, two regions with the same rainfall can have different local water management systems and thus the perceived water availability can be driven by the existence of reservoir lakes or other systems to buffer rain shortages. Secondly, a reason could be found in the circumstance that Bavaria is quite urbanized, and a high level of infrastructure is available. Therefore, inhabitants can easily travel throughout the state. Residents living in an agricultural area, for example, are likely to work in a city and spend recreational time in natural areas. It could thus be, that the land cover in a person’s location of residence does not shape their preferences and perceptions regarding ES since they also experience influences from regions with a different prevailing land cover. For example, in a study by Torralba et al. [48], values and ES attitudes differed between study sites which were all dominated by wood-pasture. Quintas-Soriano et al. [15] found a high attributed importance of cultural services irrespective of how urbanized the study site was. Thirdly, recent developments in the media and public attention could drive perceptions of respondents more than their personal experiences in their residence area does. A Bavarian referendum in 2019 aiming at strengthening biodiversity gained huge support in the population [49]. The campaign of this referendum likely increased the perceived importance of services like habitat and pollination. Also, climate change as a framing concept of ES has gained public awareness in the last years. In Germany, 81% think that climate change is a very serious problem [50] which likely means also perceiving ES like local and global climate regulation as important. In this sense, media coverage and the generally strong momentum for environmental-related topics could be another reason why the land cover and climate gradient did not show an influence on perceptions.

### 4.3 Methodological considerations and limitations

This analysis evaluates the perceived importance of ES that was originally measured on a 5-point Likert-type scale as a numeric measure when reporting the mean perceived importance per ES and when conducting the Generalized Linear Models. There is some discussion regarding which procedures are appropriate for data obtained from Likert scales [51]. Strictly speaking, the scale does only imply an order among items but it is not inherent that the intervals between the response options are equal [52]. However, other researchers speak in favour of assuming an interval scale [53] and recent research on the socio-cultural valuation of ES has been measured on Likert-scales and further on been treated as continuous [19, 24, 42]. It was decided to handle the response variables as continuous for the analysis in the present study. Factors for this decision were firstly, that in the survey the response scale was framed with signs ranging from “- -” to “+ +” along with the verbal statement “very unimportant” to “very important”. This can be seen as an implication for the respondents that the scale is continuous. Secondly, some concerns raised about treating Likert-type data continuously did not seem applicable in this specific case. For example, it was mentioned the calculated mean might be distorted if the answers centre around the negative and positive ends of the scale, resulting in a neutral mean value [54]. In this study however, it can be seen in Fig 4 that the vast majority of answers is located on the positive side of the scale. Therefore, calculating the mean from these answers does not seem prone to this distorting effect.

Overall, scores on the Likert-scale given to single ES were high, making it harder to see clear preferences among respondents. This is a finding which also resulted from similar studies [42]. While many ES are probably indeed highly recognized by respondents, the high scores might also partly stem from the survey context where respondents are not facing an actual trade-off situation [31]. Using a point allocation method instead would be a possibility to avoid this by urging respondents to assign a ranking order, e.g. by asking them to award a total of 100 points among a list of ES [55].

The study sample was not representative (see S8 Table in S1 File). Participants were more often male with an above average age and educational background and the societal group of farmers is an over proportional part of the sample. This fact might bias the results towards the answer pattern of farmers if not distinguishing between societal actor groups. Moreover, a certain self-sampling effect can be expected. When receiving a survey invitation by email letter or through a newspaper article, naturally, people with a higher affinity for the promoted topic will participate. Thus, the respondents are likely persons generally interested in topics related to ecosystems and nature conservation. Out of the citizens even 271 were engaged with nature and landscape in their activities (full/half time occupation or honorary activity). This is also shown by the stated interest in further contacts. Overall, 59% of participants were interested in survey results, 31% in further surveys, and 12% in workshops on the topic. Though, differences between groups existed, e.g. 71% of citizens, 48% of farmers, 74% of foresters and 72% nature managers were interested in the survey results and received a short report via email.

In order to further explore the cultural context of the different societal actor groups it is advisable to investigate in future surveys the participants’ worldviews, knowledge and values, which are all influencing the perception of and decisions on ecosystem services [56]. Capturing the previous experiences of the societal groups would also add to the understanding of current observations related to ecosystem services. In our study we strictly investigated individuals and assumed that group preferences were built bottom-up as the sum of the individual preferences. Investigating group preferences e.g. via content analysis of published statements of organization would be an interesting extension to our study.

## 5. Conclusion

This study contributes to an understanding of societal perceptions of ecosystem services in Bavaria. It has demonstrated the overall high perceived importance of ES across all societal actor groups. For some ES, differences existed between the societal actors. Farmers rated some provisioning services higher than the other societal actors, especially energy plant production. For several regulating and cultural services, farmers rated these slightly lower than citizens. The preservation of soil fertility and protection of groundwater quality received the highest perceived importance across groups, while production of energy plants obtained the lowest. The hypothesized influence of climate and land cover gradients on ES perception only held true in two cases. Living in a near-natural region decreased the attributed importance to energy plant production whereas mean annual precipitation increased the perceived importance of animal production in the open field.

Strengths of this research are the large-scale data collection across Bavaria and the targeted sampling of four different societal actor groups. The questionnaire targeting 21 ES allowed for a comprehensive insight into ES valuation and perception. Depending on the research interest, the results can be examined either on Bavarian level or broken down to a more local level. Moreover, these results contribute to an integrated socio-cultural valuation, counteracting the majority of biophysical and monetary ES valuation studies [4]. However, it could be interesting to combine the socio-cultural valuation of ES from this study with biophysical data on the supply of ES in Bavaria.

Commonly mentioned benefits resulting from research on ES is science-based knowledge accumulation, enhanced participation and collaboration, as well as awareness raising [57, 58]. This study has revealed the views of four societal actors in Bavaria on a comprehensive set of ES. Knowing about stakeholders’ preferences is a first important step to provide implications for adequate land use management. Knowing attitudes and preferences towards ES is especially important for decision and policies in environmental planning since people’s support is a fundamental prerequisite for successful implementation [59].

## Supporting information

S1 File Supporting information containing (S1-S41 Figs) and (S1-S9 Tables). (DOCX)
