# Supplementary material for: Perceptions of ecosystem services: Comparing socio-cultural and environmental influences
Source: PLoS One. 2022 Oct 27;17(10):e0276432. doi: 10.1371/journal.pone.0276432 (PMC9612508; doi:10.1371/journal.pone.0276432)
Supplement: S1 File — (DOCX) [file pone.0276432.s001.docx]

Supporting information for

**Perceptions of ecosystem services: comparing socio-economic and environmental explanatory factors**

Miriam Thiemann^1^, Rebekka Riebl^1^, Maria Haensel^1^, Thomas M. Schmitt^1*^, Manuel J. Steinbauer^2^, Theresa Landwehr^1^, Ute Fricke^3^, Sarah Redlich^3^ and Thomas Koellner^1^

^1^ University of Bayreuth, Professorship of Ecological Services, Bayreuth Center of Ecology and Environmental Research (BayCEER), Universitätsstraße 30, 95447 Bayreuth, Germany

^2^ University of Bayreuth, Sports Ecology, Bayreuth Center of Ecology and Environmental Research (BayCEER) & Bayreuth Center of Sport Science (BaySpo), Universitätsstraße 30, 95447 Bayreuth, Germany

^3^ Department of Animal Ecology and Tropical Biology, Julius-Maximilians-University, 97074 Würzburg, Germany

Detailed information for the sampling process

*Table S1. A) Targeted postal delivery districts with their numbers of households, quadrant type (climate and land cover gradients) based on https://postaktuell-manager.deutschepost.de/PostaktuellFrontend/ (total n = 44,244) and B) successfully contacted local newspapers who promoted the LandKlif survey with date of publication.*

| A) |  |  |  |
| --- | --- | --- | --- |
| Quadrant type | Address | Postal delivery district | No. of households |
| 1_nature | Bogenbergsweg, Bischofswiesen | 83471-24 | 298 |
|  |  | 83471-26 | 359 |
|  |  | 83471-27 | 419 |
|  |  | 83471-30 | 373 |
|  |  | 83471-31 | 513 |
|  |  | 83471-44 | 493 |
|  |  | 83471-45 | 321 |
|  |  | 83483-33 | 347 |
|  |  | 83483-41 | 437 |
|  |  | 83483-43 | 408 |
| 2_nature | Schneewinklweg, Anger | 83451-34 | 336 |
|  |  | 83454-29 | 443 |
|  |  | 83454-31 | 387 |
|  | Prüllsbirkig BY | 91278-21 | 444 |
|  |  | 91278-24 | 573 |
| 3_nature | Ebersberg BY | 85560-01 | 344 |
|  |  | 85560-03 | 616 |
|  |  | 85560-80 | 164 |
|  |  | 85560-81 | 547 |
|  |  | 85560-82 | 359 |
|  |  | 85560-83 | 329 |
|  |  | 85560-84 | 340 |
|  |  | 85560-85 | 380 |
|  |  | 85643-90 | 420 |
| 4_nature | Steintalerhof Lohr a. Main | 97816-01 | 814 |
|  |  | 97816-03 | 713 |
|  |  | 97816-09 | 499 |
|  |  | 97816-23 | 646 |
|  |  | 97816-02 | 664 |
|  |  | 97833-12 | 565 |
| 5_nature | Kleinwernfeld BY | 97737-01 | 640 |
|  |  | 97737-06 | 549 |
|  |  | 97737-07 | 592 |
|  |  | 97737-08 | 602 |
| 1_agriculture | Stegenwaldhauser Str. Selbitz | 95152-11 | 437 |
|  |  | 95152-71 | 556 |
|  |  | 95152-74 | 465 |
|  |  | 95189-91 | 535 |
|  | Posterlitz Schwarzenbach a.d. Saale | 95126-71 | 522 |
|  |  | 95126-72 | 602 |
|  |  | 95176-83 | 407 |
| 2_agriculture | Steinrißl 15 Germaringen | 86860-41 | 528 |
|  |  | 87656-72 | 465 |
|  |  | 86860-42 | 343 |
|  |  | 86869-61 | 349 |
|  | Mannsdorf BY (Parsberg) | 92331-06 | 361 |
|  |  | 92331-10 | 229 |
|  |  | 92363-23 | 459 |
| 3_agriculture | Lerchen 5 Haarbach | 94542-70 | 340 |
|  |  | 84385-82 | 382 |
|  | Burgleite Greding | 91171-53 | 537 |
|  |  | 91171-55 | 473 |
|  | Grafendorfer Straße Sielstetten | 85413-31 | 348 |
|  |  | 84104-41 | 477 |
| 4_agriculture | Barhof 1 Ruhstorf an der Rott | 94099-20 | 385 |
|  |  | 94099-21 | 421 |
|  | Fährbrückerstr Hausen b Würzburg | 97262-16 | 426 |
|  |  | 97262-17 | 451 |
| 5_agriculture | Bruckhof Pocking | 94060-09 | 523 |
|  |  | 94060-11 | 363 |
|  |  | 94099-16 | 342 |
|  |  | 94152-22 | 328 |
|  | Daimlerstraße Waldbrunn | 97249-08 | 560 |
|  |  | 97295-21 | 547 |
|  |  | 97295-22 | 669 |
|  |  | 97297-23 | 525 |
| 1_urban | Lindenfeld Weg 28 Grassau | 83224-19 | 200 |
|  |  | 83224-23 | 583 |
|  |  | 83224-24 | 509 |
|  |  | 83224-25 | 418 |
|  |  | 83250-28 | 449 |
|  |  | 83250-29 | 448 |
| 2_urban | Saliterstr. 32 Marktoberdorf | 87616-02 | 632 |
|  |  | 87616-05 | 716 |
|  |  | 87616-06 | 678 |
|  |  | 87616-12 | 598 |
|  |  | 87616-13 | 939 |
|  |  | 87616-17 | 420 |
| 3_urban | Auenweg 1 Traunstein | 83278-03 | 356 |
|  |  | 83278-04 | 398 |
|  |  | 83278-05 | 459 |
|  |  | 83278-21 | 240 |
|  |  | 83278-27 | 215 |
|  |  | 83278-28 | 359 |
|  |  | 83278-29 | 424 |
| 4_urban | Stallerweg 10 Passau | 94036-37 | 442 |
|  |  | 94036-38 | 542 |
|  |  | 94036-42 | 1 |
|  |  | 94036-42 | 641 |
|  |  | 94036-45 | 755 |
| 5_urban | Salierstraße 1 Bamberg | 96049-65 | 340 |
|  |  | 96049-80 | 388 |
|  |  | 96049-81 | 539 |
|  |  | 96049-82 | 454 |
|  |  | 96049-83 | 96 |
|  |  | 96049-83 | 361 |
|  |  | 96049-98 | 355 |

| B) Name of the newspaper | Date |
| --- | --- |
| 1. Nordbayerischer Kurier Bayreuth | 18.04.2020 |
| 1. Passauer Neue Presse – Passau Stadt | 28.04.2020 |
| 1. Allgäuer Zeitung Füssen | 05.05.2020 |
| 1. Allgäuer Zeitung Kaufbeuren | 07.05.2020 |
| 1. Schwabacher Tageblatt | 16.05.2020 |
| 1. Wochenblatt Traunstein Berchtesgadener Land | 16.05.2020 |
| 1. Allgäuer Zeitung Marktoberdorf | 17.05.2020 |
| 1. Frankenpost Hof | 18.05.2020 |
| 1. Moosburger Zeitung – Erding Freising | 19.05.2020 |
| 1. Erdinger Wochenblatt | 20.05.2020 |
| 1. Freisinger Wochenblatt | 20.05.2020 |
| 1. Pegnitz Zeitung – Nürnberger Land | 21.05.2020 |
| 1. Bayreuther Tagblatt | 22.05.2020 |
| 1. Pressestelle Landkreis Freyung-Grafenau | 22.05.2020 |
| 1. Landratsamt Bamberg | 26.05.2020 |
| 1. Main-Echo Lohr | 26.05.2020 |
| 1. Mittelbayerische Zeitung – Neumarkt-Parsberg | 26.05.2020 |
| 1. Neumarkter Tagblatt | 26.05.2020 |
| 1. Passauer Neue Presse – Regen | 27.05.2020 |
| 1. Frankenpost Marktredwitz | 28.05.2020 |
| 1. Main Post Main Spessart | 28.05.2020 |
| 1. Roth-Hilpoltsteiner Volkszeitung | 28.05.2020 |
| 1. Bayreuther Sonntagszeitung | 07.06.2020 |
| 1. Main-Post Würzburg | 13.06.2020 |
| 1. Traunsteiner Tagblatt | 13.06.2020 |

*Table S2. List of ÄELF (agencies for nutrition, agriculture, and forestry) where farmers were approached to fill out surveys on tablets.*

| Bayreuth | Münchberg - Wunsiedel |
| --- | --- |
| Bamberg | Passau |
| Ebersberg | Regen – Regen |
| Erding | Regen – Waldkirchen |
| Karlstadt – Karlstadt | Roth – Roth |
| Karlstadt – Aschaffenburg | Roth - Hersbruck |
| Kaufbeuren | Traunstein |
| Münchberg - Münchberg | Würzburg |


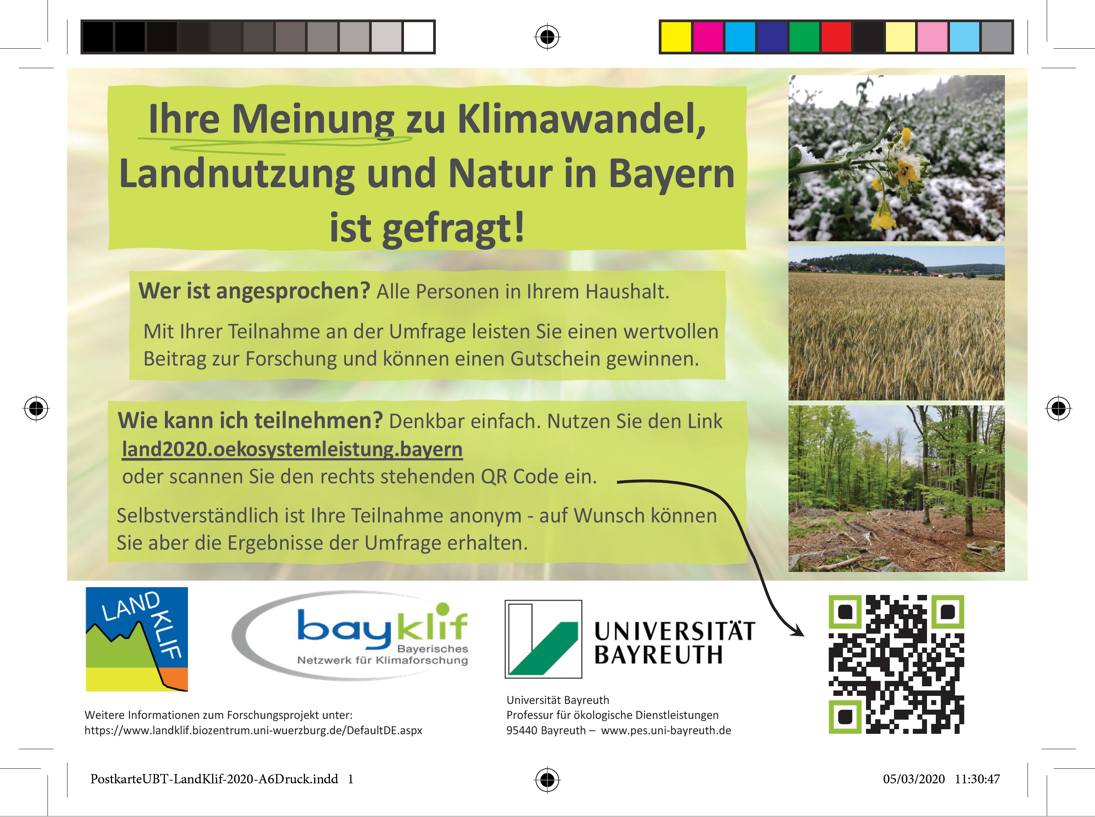


*Figure S1: Postcard sent as invitation to 44,244 households in Bavaria.*Questionnaire items and structure

*Table S3. Questions from the LandKlif questionnaire used for the analysis.*

| Question | Response categories |
| --- | --- |
| Which gender do you have? | - Male - Female - Divers - No answer |
| Which age category do you belong to? | <18, 18-25, 26-30, 31-35, 36-40, 41-45, 46-50, 51-55, 56-60, 61-65, 65-70, >70 |
| For which private activities do you use nature and landscapes in Bavaria? Please choose (Multiple answers possible) | - Walking, hiking, running, or biking - Observing wildlife - Collecting mushrooms, wild herbs or berries - Trips by car or motorcycle - Fishing or hunting - Other (please indicate): - No private activities |
| Which general education degree did you obtain? Please only indicate the highest degree. | - Lower secondary school - Middle secondary school - Higher secondary school - Polytechnic secondary school - Without general education degree - No answer |
| Did you already hear of the term ecosystem services? | - Yes - No |
| Please fill in the zip code of your residence: | < free> |
| How important do you think are the following services of nature and landscapes in all of Bavaria? Please click.   - < See list of 21 services as presented in the questionnaire below. > | - Very unimportant - Unimportant - Indifferent - Important - Very important - I don’t know |

*Table S4. List of ecosystem services in the survey. Categorization in comparison to the classifications of TEEB (2010) and Rabe et al. (2016).*

|  | **LandKlif Survey** | **Rabe et al. 2016** | **TEEB service** |
| --- | --- | --- | --- |
| Provisioning services | Production of food plants | Crop capacity | Agricultural products |
|  | Fodder production on permanent grasslands |  |  |
|  | Animal production in the open field |  |  |
|  | Mushrooms, berries, and herbs |  | Collectible products |
|  | Timber production in forests |  | Wood |
|  | Production of energy plants |  | Biomass fuel |
| Regulating services | Pollination of crop plants |  | Pollination |
|  | Biological pest control |  | Pest regulation |
|  | Soil erosion reduction | Wind and Water Erosion Prevention | Wind and Water erosion prevention |
|  | Preservation of soil fertility |  | Soil formation, soil fertility |
|  | Protection of groundwater quality | Water Quality Regulation | Water quality regulation |
|  | Groundwater formation |  | Groundwater recharge, |
|  | Flood control |  | Flood control |
|  | Air purification | Air Quality Regulation | Air quality regulation by vegetation |
|  | Local climate regulation |  | Regional climate regulation |
|  | Global climate regulation | Carbon storage | Carbon storage |
| Cultural services | Recreation in urban areas |  | Recreation in public space (settlement) |
|  | Recreation in open landscapes | Recreation Protected Areas | Recreation in open landscape |
|  | Sense of place |  | Spiritual experience, sense of place |
|  | Learning and inspiration |  |  |
| Habitat services | Habitat for wildlife and wild plants |  | Lifecycle maintenance, habitat and gene pool protection |

*Table S5. List of the 21 ecosystem services as written out fully in the questionnaire in English and German (original).*

| **Provisioning Services** |
| --- |
| - Production of energy plants (e.g. rape, corn, Chinese reed) - Produktion von Energiepflanzen im Ackerbau (z.B. Raps, Mais, Chinaschilf) |
| - Animal production in the open field (e.g. milk cows and fattening of bullocks on pastures) - Tierproduktion im Freiland (z.B. Milchkühe und Ochsenmast auf Weiden) |
| - Mushrooms, berries, and herbs - Pilze, Beeren und Kräuter |
| - Production of food plants (e.g. grain, oilseeds, vegetables) - Produktion von Nahrungspflanzen im Ackerbau (z.B. Getreide, Ölsaaten, Feldgemüse) |
| - Production of fodder on permanent grasslands (e.g. grass silage and hey) - Produktion von Tierfutter im Dauergrünland (z.B. Grassilage, Heu) |
| - Groundwater formation (through an increased percolation rate in grasslands and forests) - Grundwasserneubildung (durch Erhöhung der Sickerrate im Grünland und Wald) |
| - Timber production in the forests - Holzproduktion im Wald |
| **Regulating Services** |
| - Protection of groundwater quality (e.g. by nutrient retention in vegetation and soil) - Schutz der Grundwasserqualität (z.B. durch Nährstoffrückhalt in der Vegetation und Boden) |
| - Flood protection (e.g. through meadows and floodplains) - Hochwasserschutz (z.B. durch Wiesen und Auenbereiche) |
| - Regulating the global climate (by uptake of climate gases as CO2 and storage in soils and vegetation) - Regulation des globalen Klimas (durch Aufnahme von Klimagasen wie CO2 und Speicherung in Böden und Vegetation) |
| - Regulating the local climate (by shadowing, evaporation of water and formation of cold-air corridors) - Regulation des lokalen Klimas (durch Schattenwurf, Verdunstung von Wasser und Bildung von Kaltluftschneisen) |
| - Biological pest control (through wild insects and birds) - Biologische Schädlingskontrolle (durch wildlebende Insekten und Vögel) |
| - Air purification (Vegetation filters the air and reduces e.g. particulate matter concentration) - Luftfilterung (Filterung der Luft durch Vegetation und dadurch Reduktion z.B. von Feinstaubbelastung) |
| - Decreased soil erosion (e.g. by vegetation) - Minderung der Bodenerosion (z.B. durch Vegetationsdecke) |
| - Preservation of soil fertility (by natural soil processes) - Erhalt der Bodenfruchtbarkeit (durch natürliche Bodenprozesse) |
| - Pollination of crop plants (by wild insects) - Bestäubung von Nutzpflanzen (durch wildlebende Insekten) |
| **Cultural Services** |
| - Sense of place (landscapes that contribute to sense of identity) - Vertrautheit und Heimat (durch identitätsstiftende Landschaften) |
| - Outdoor recreation (through nature experiences and aesthetical landscapes) - Erholung in der freien Landschaft (durch Naturerleben und Landschaftsästhetik) |
| - Recreation in urban areas (through parks and other green space areas) - Erholung im öffentlichen Siedlungsraum (durch Parkanlagen und andere Grünflächen) |
| - Learning and inspiration (in and through nature) - Lernen und Inspiration (in der und durch die Natur) |
| **Habitat Services** |
| - Habitat for wildlife and wild plants (e.g. birds, insects, mammals, wildflowers) - Lebensraums für wildlebende Tier- und Pflanzenarten (z.B. Vögel, Insekten, Säugetiere, Wildblumen) |

*Table S6. Division of the 21 ecosystem services into one random and three steady subsets.*

| **Subset full** | Random 7 out of 21 ES |
| --- | --- |
| **Subset 1** | Production of food plants, animal production in the open field, non-timber forest products, biological pest control, global climate regulation, recreation in urban areas, learning and inspiration |
| **Subset 2** | Fodder production, pollination, protection of groundwater quality, groundwater formation, air purification, sense of place, recreation in open landscapes |
| **Subset 3** | Production of timber, production of energy plants, soil erosion reduction, preservation of soil fertility, flood control, local climate regulation, habitat |

Descriptive results of sample


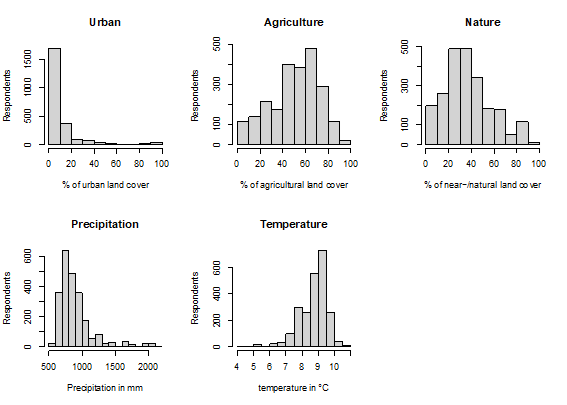


Figure S2. Distribution of number of respondents along the gradients of climate and land use.

*Table S7. Distribution of socio-economic features by societal actors. Survey percentages refer to the total of people who answered the question respectively; missing values excluded.*

|  | | All | Citizens | Farmers | Foresters | Nature  managers | Bavaria |
| --- | --- | --- | --- | --- | --- | --- | --- |
| No. of respondents | | 3,018 | 948 | 1676 | 225 | 169 | 13 million |
| Gender (%) | Male  Female | 73.3  26.7 | 51.6  48.4 | 85.9  14.1 | 90.1  9.9 | 59.2  40.8 | 49.6  50.4 |
| Age (%) | < 18 | 0.7 | 1.6 | 0.2 | 0.5 | 0 | 16.4 |
|  | 18-25 | 3.9 | 6.3 | 2.8 | 2.6 | 2.7 | 9.2 |
|  | 26-30 | 5.2 | 4.0 | 4.5 | 6.7 | 16.4 | 6.8 |
|  | 31-35 | 7.9 | 8.4 | 7.4 | 8.7 | 8.9 | 6.6 |
|  | 36-40 | 8.5 | 7.0 | 9.7 | 8.2 | 6.8 | 6.4 |
|  | 41-45 | 9.9 | 8.2 | 11.1 | 10.8 | 6.8 | 6 |
|  | 46-50 | 13.2 | 10.1 | 15.7 | 10.8 | 10.3 | 7.3 |
|  | 51-55 | 16.7 | 15.1 | 18.2 | 13.8 | 14.4 | 8.3 |
|  | 56-60 | 13.7 | 13.3 | 13.4 | 15.9 | 16.4 | 7.5 |
|  | 61-65 | 11.2 | 11.5 | 10.7 | 15.9 | 8.2 | 6.0 |
|  | 66-70 | 5.3 | 7.6 | 4.2 | 3.6 | 4.8 | 4.3 |
|  | > 70 | 3.7 | 7.0 | 1.9 | 2.6 | 4.1 | 15.0 |
| Education^1)^ (%) | Lower | 21.5 | 7.0 | 34.6 | 6.2 | unclear | 49.9 |
|  | Middle | 28 | 26.5 | 33.9 | 13 | unclear | 24.0 |
|  | Higher | 50.4 | 66.6 | 31.6 | 80.8 | 86.0 | 26.1 |
| Knowledge of ES term (%) | Yes | 36.7 | 23.6 | 35.3 | 63.3 | 89.0 | NA |
|  | No | 63.3 | 76.4 | 64.7 | 36.7 | 11.0 | NA |
| No. of outdoor activities | 0 | 2.5 | 0.6 | 3.9 | 1.3 | 0.6 | NA |
|  | 1 | 28.1 | 28.5 | 30.9 | 14.3 | 17.2 | NA |
|  | 2 | 33.0 | 32.6 | 34.8 | 29.5 | 22.5 | NA |
|  | 3 | 23.0 | 26.0 | 20.8 | 28.1 | 39.6 | NA |
|  | 4 | 10.1 | 10.8 | 7.9 | 19.2 | 15.4 | NA |
|  | 5 | 2.1 | 1.3 | 1.8 | 5.8 | 4.1 | NA |
|  | 6 | 0.2 | 0.2 | 0 | 1.8 | 0.6 | NA |

^1)^ Education level according to German systems:

Lower: Ohne allgemeinen Schulabschluss; Haupt-(Volks-)Schulabschluss

Middle: Realschul- oder gleichwertiger Abschluss

Higher: Fachhochschul- oder Hochschulreife; Abschluss der polytechnischen Oberschule

**

*Figure S3: Histogram of the duration of the respondents’ participation in the survey. In the introduction of the survey, 20 minutes were announced.*

Data preparation

*Table S8. Aggregation of land cover categories from CORINE 2018 to the main land cover categories used in the analysis.*

| **Aggregated category** | **Original categories in CORINE Land Cover 2018** |
| --- | --- |
| Urban | Continuous urban fabric  Discontinuous urban fabric  Industrial or commercial units  Road and rail networks and associated land  Port areas  Airports  Mineral extraction sites  Dump sites  Construction sites  Green urban areas  Sport and leisure facilities |
| Agricultural | Non-irrigated arable land  Permanently irrigated land  Rice fields  Vineyards  Fruit trees and berry plantations  Olive groves  Pastures  Complex cultivation patterns  Land principally occupied by agriculture with significant areas of natural vegetation  Agro-forestry areas |
| Semi-natural | Broad-leaved forest  Coniferous forest  Mixed forest  Natural grasslands  Moors and heathland  Sclerophyllous vegetation  Transitional woodland-shrub  Beaches, dunes, sands  Bare rocks  Sparsely vegetated areas  Burnt areas  Glaciers and perpetual snow  Inland marshes  Peat bogs  Salt marshes  Salines  Interdial flats  Water courses  Water bodies  Coastal lagoons  Estuaries  Sea and ocean |


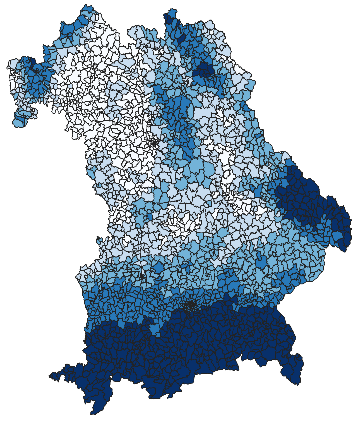

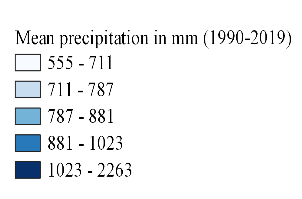

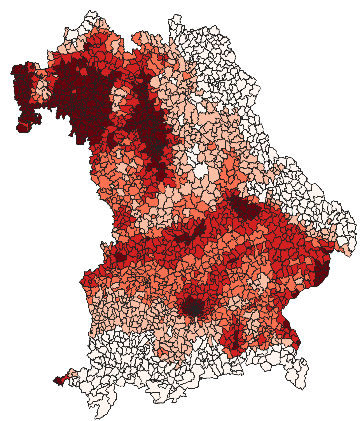

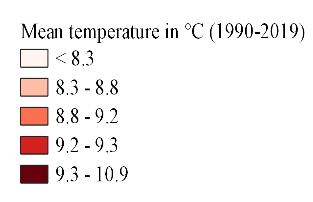

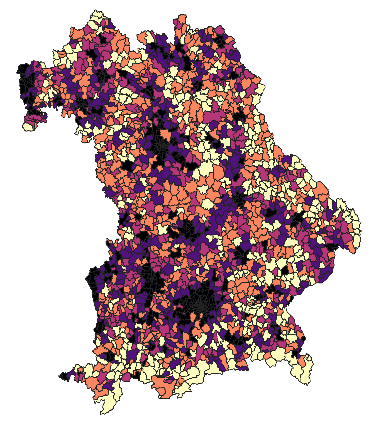

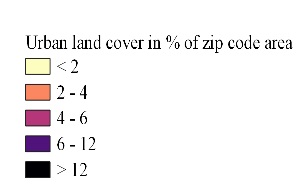

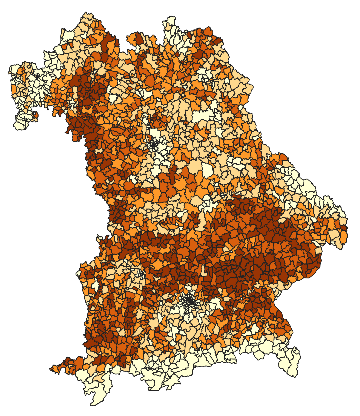

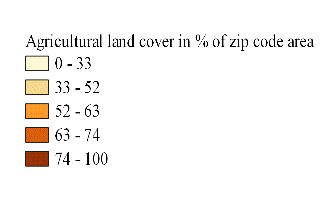

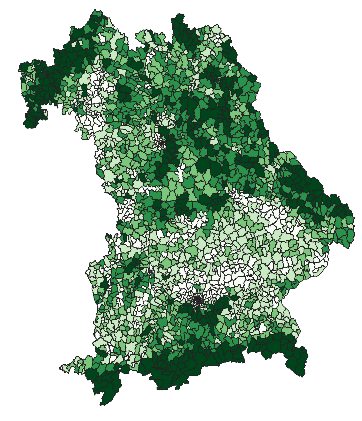

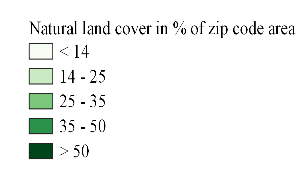


*Figure S4. Maps of distribution of gradient variables across Bavaria.*

Table S9. Conducted tests per explanatory variable and ecosystem service with p-values. Numbers in grey indicate insignificance.

Likert plots by categorical explanatory variables for all 4 sets

*Figure S5. Perceived importance of ecosystem services by gender.*

**

*Figure S6. Perceived importance of ecosystem services by completed school degree.*

*Figure S7. Perceived importance of ecosystem services of people who know the term ecosystem service vs. those who do not.*

*Figure S8. Perceived importance of ecosystem services by amount of practised outdoor hobbies. A total of six outdoor hobbies were possible to fill in the survey. However, only one person with six hobbies answered per ecosystem service.*

Figure S9. Biplots of the redundancy analysis for each of the constant ES subsets individually. The biplots show the relationship between the respective seven ecosystem services per subset and the explanatory variables, i.e. individual characteristics and gradient variables.

Response distribution per ecosystem service across Bavaria


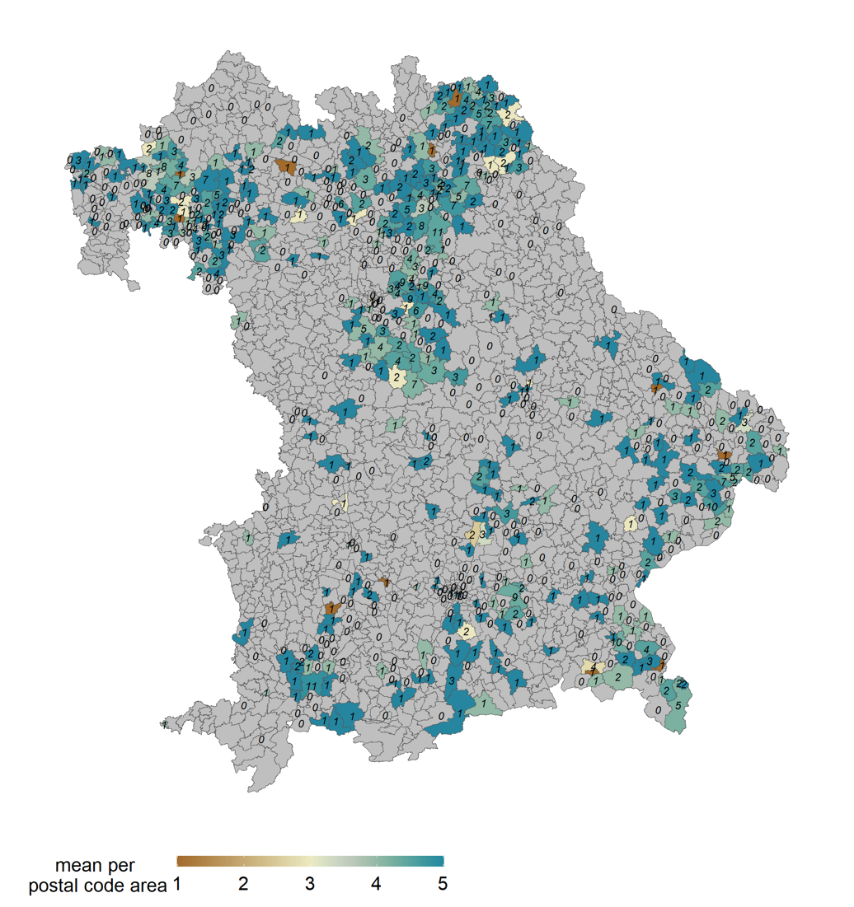


*Figure S10. Perceived importance of food plant production.* *The map shows the mean response in each postal code area. The small number within each postal code polygon represents the number of respondents from this area.*


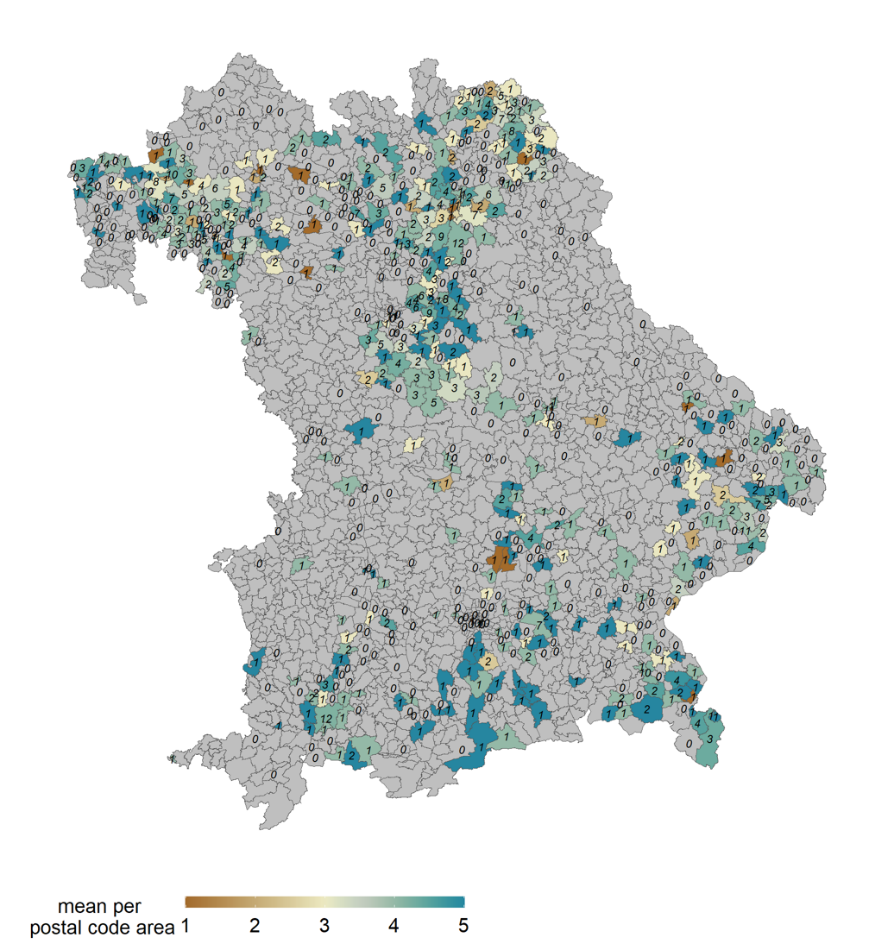


*Figure S11. Perceived importance of animal production in the open field. The small number within each postal code polygon represents the number of respondents from this area.*


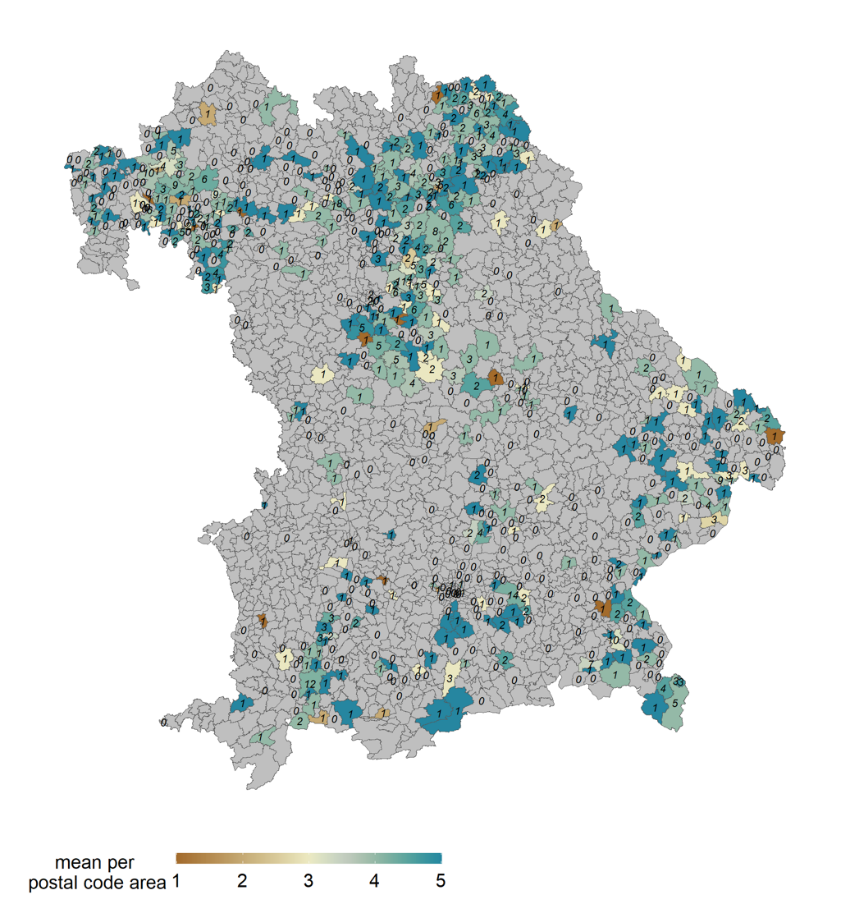


*Figure S12. Perceived importance of fodder production. The small number within each postal code polygon represents the number of respondents from this area.*

**
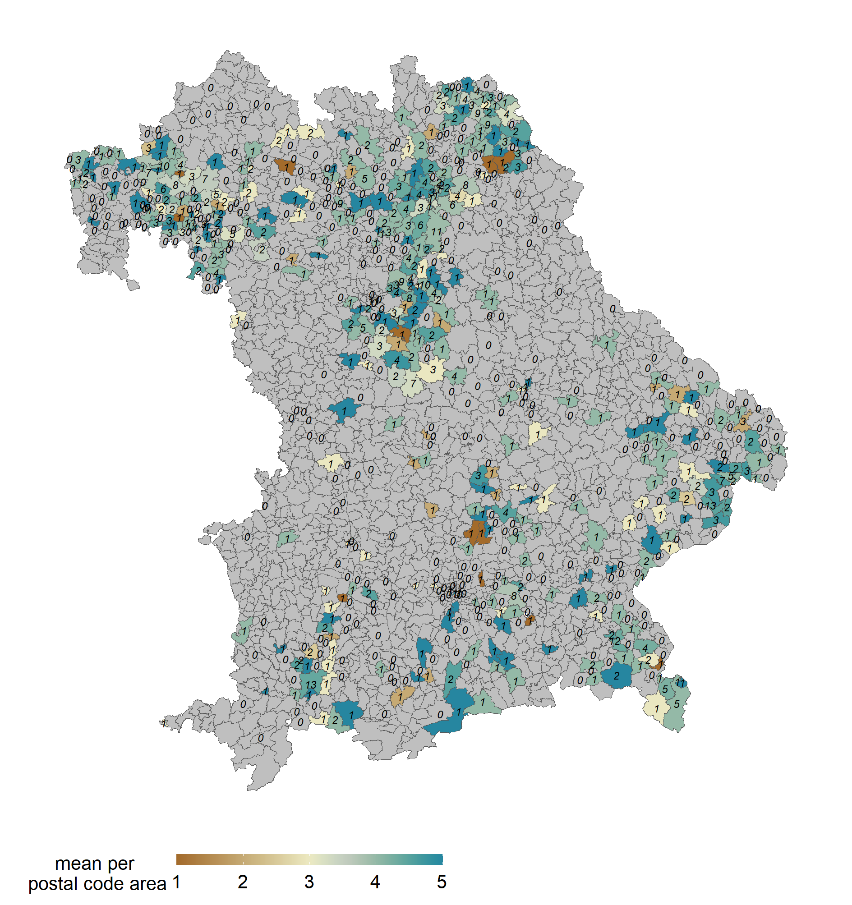
**

*Figure S13. Perceived importance of non-timber forest products. The small number within each postal code polygon represents the number of respondents from this area.*

**
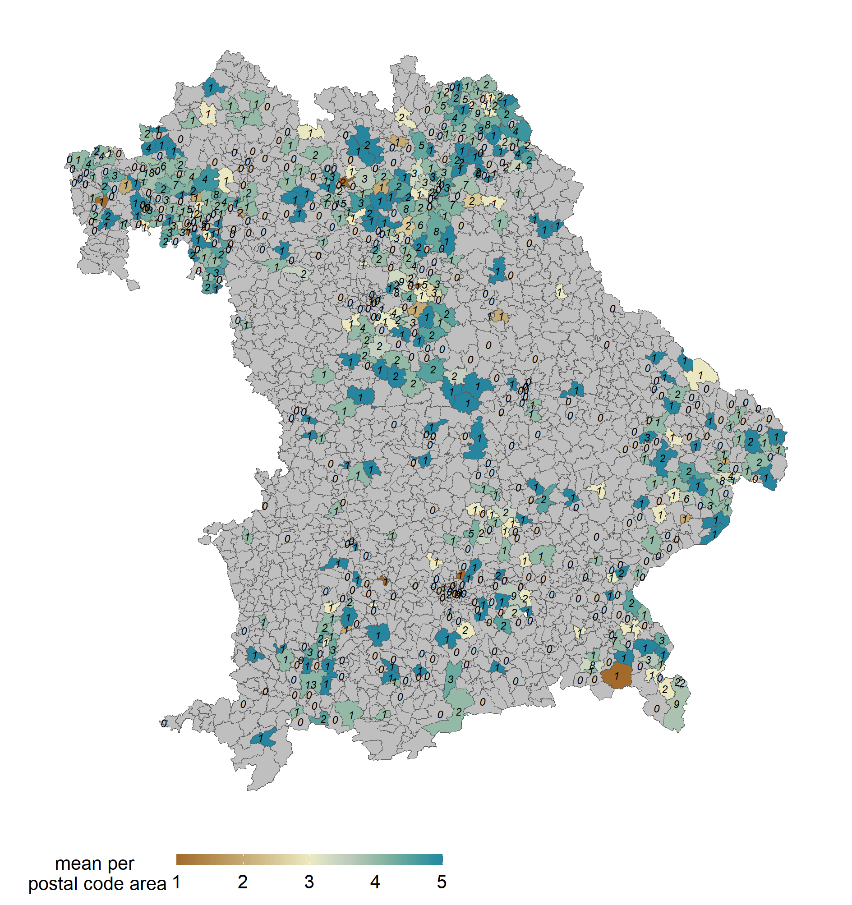
**

*Figure S14. Perceived importance of timber production. The small number within each postal code polygon represents the number of respondents from this area.*

**
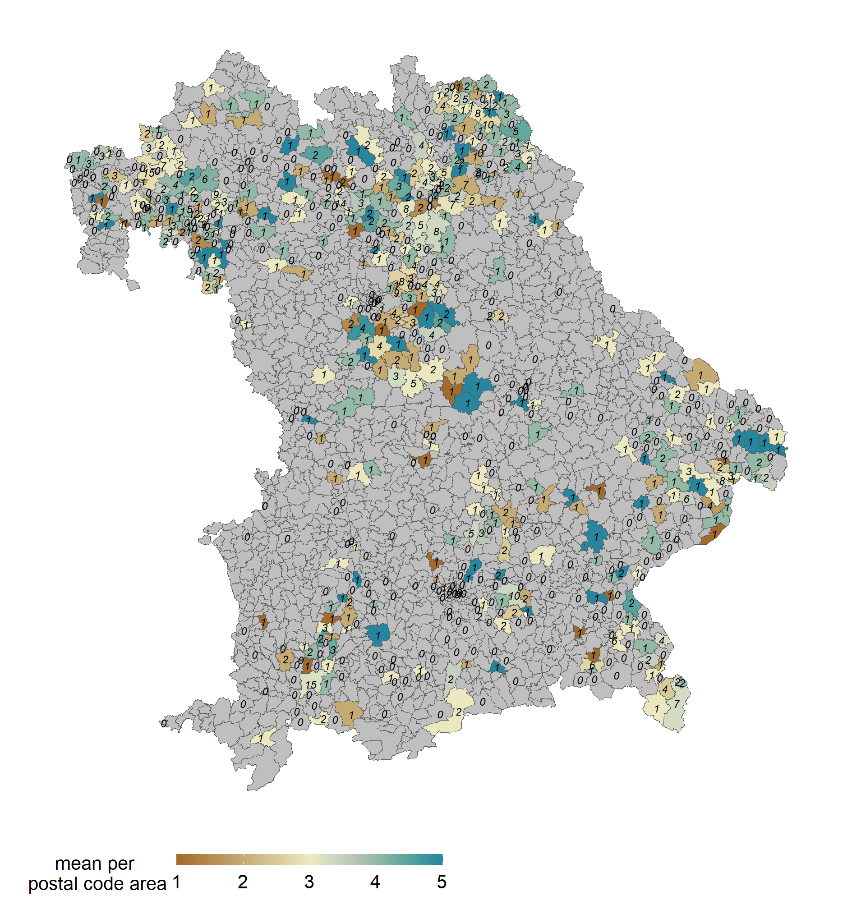
**

*Figure S15. Perceived importance of energy plant production. The small number within each postal code polygon represents the number of respondents from this area.*

**
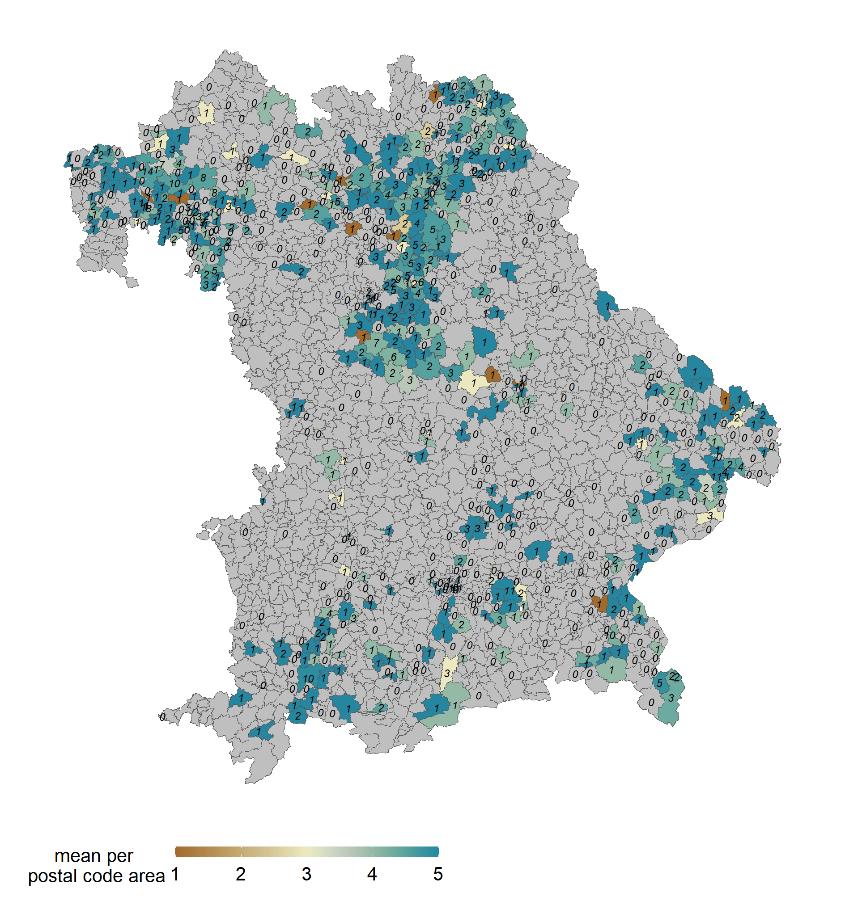
**

*Figure S16. Perceived importance of pollination. The small number within each postal code polygon represents the number of respondents from this area.*

**
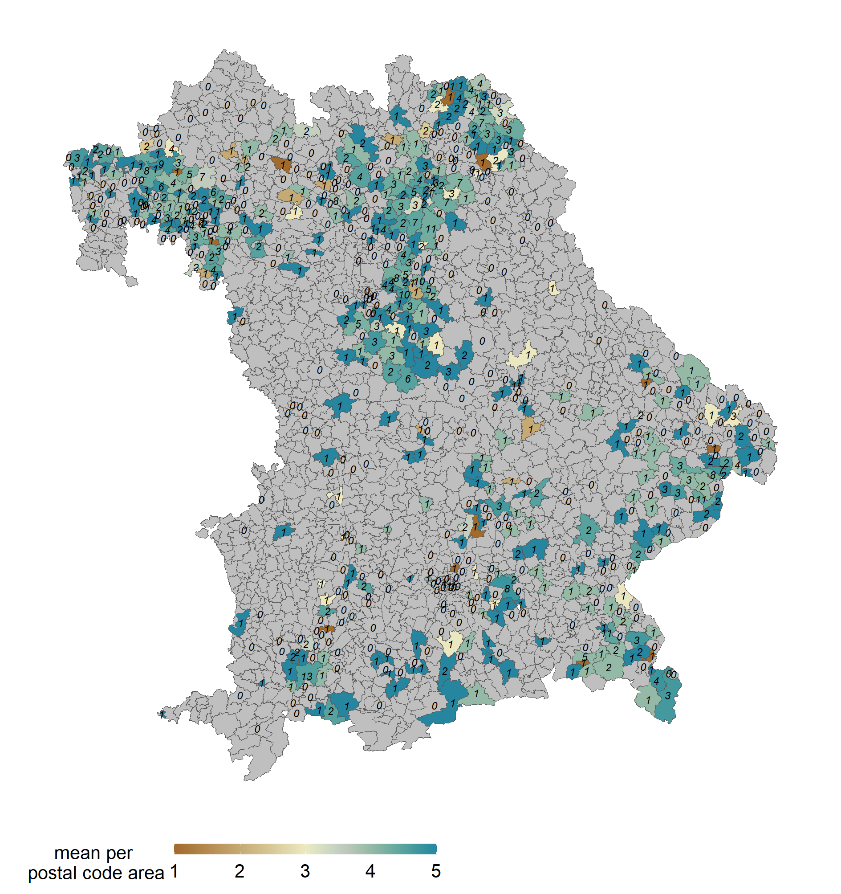
**

*Figure S17. Perceived importance of biological pest control. The small number within each postal code polygon represents the number of respondents from this area.*

**
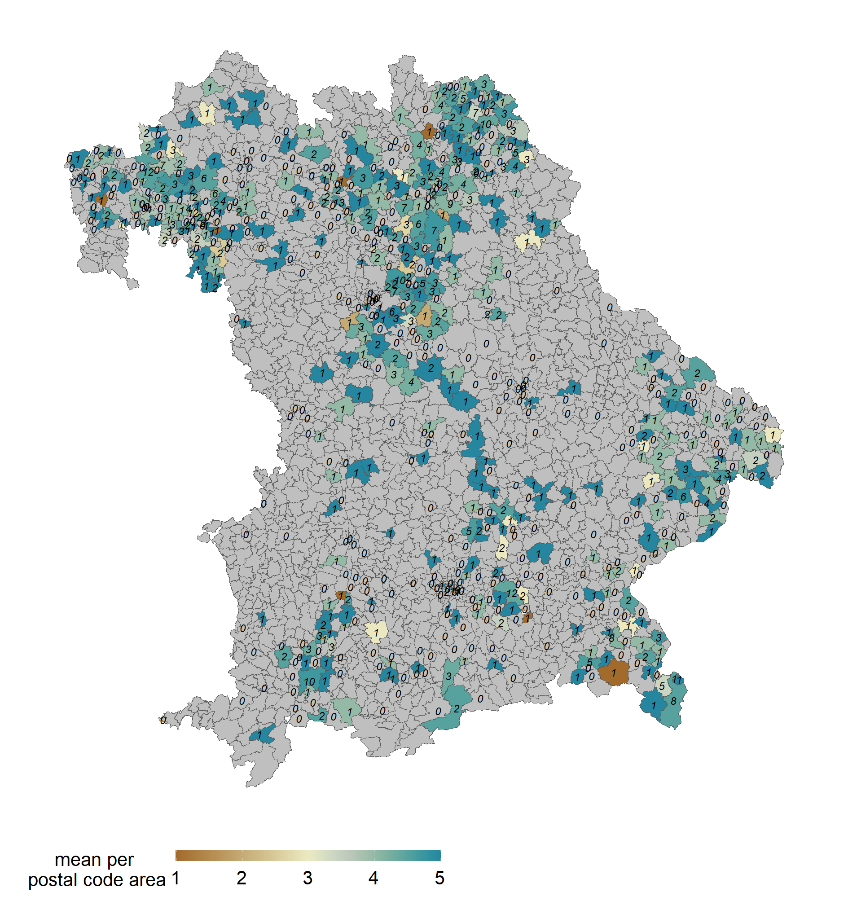
**

*Figure S18. Perceived importance of soil erosion reduction. The small number within each postal code polygon represents the number of respondents from this area.*

**
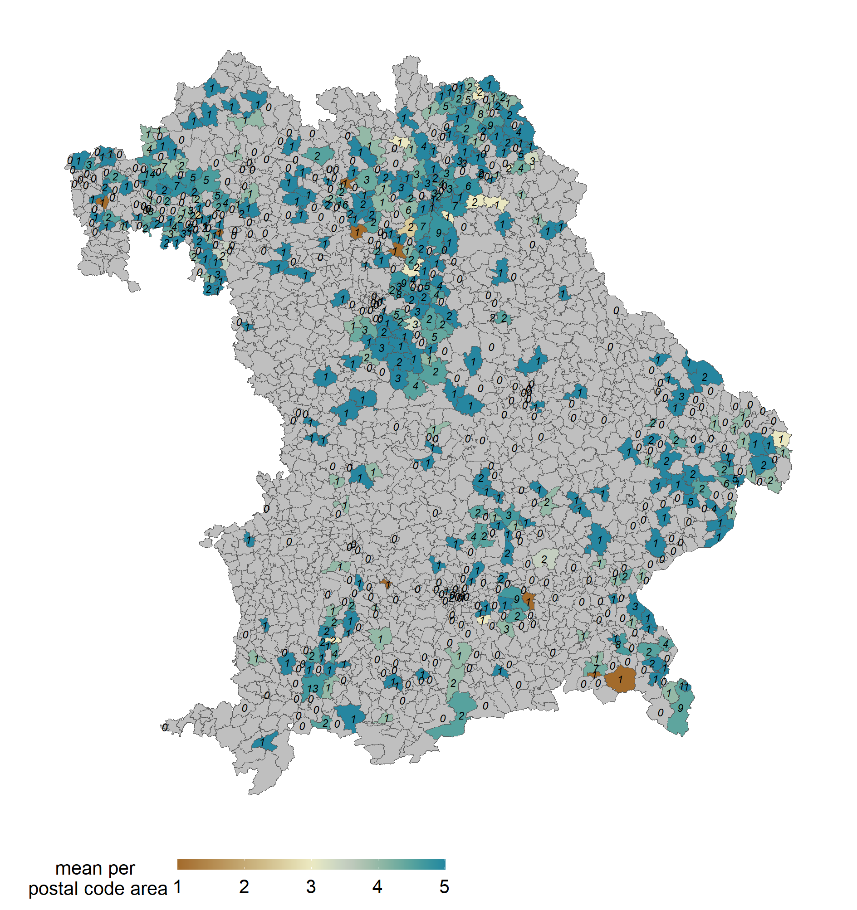
**

*Figure S19. Perceived importance of soil fertility. The small number within each postal code polygon represents the number of respondents from this area.*

**
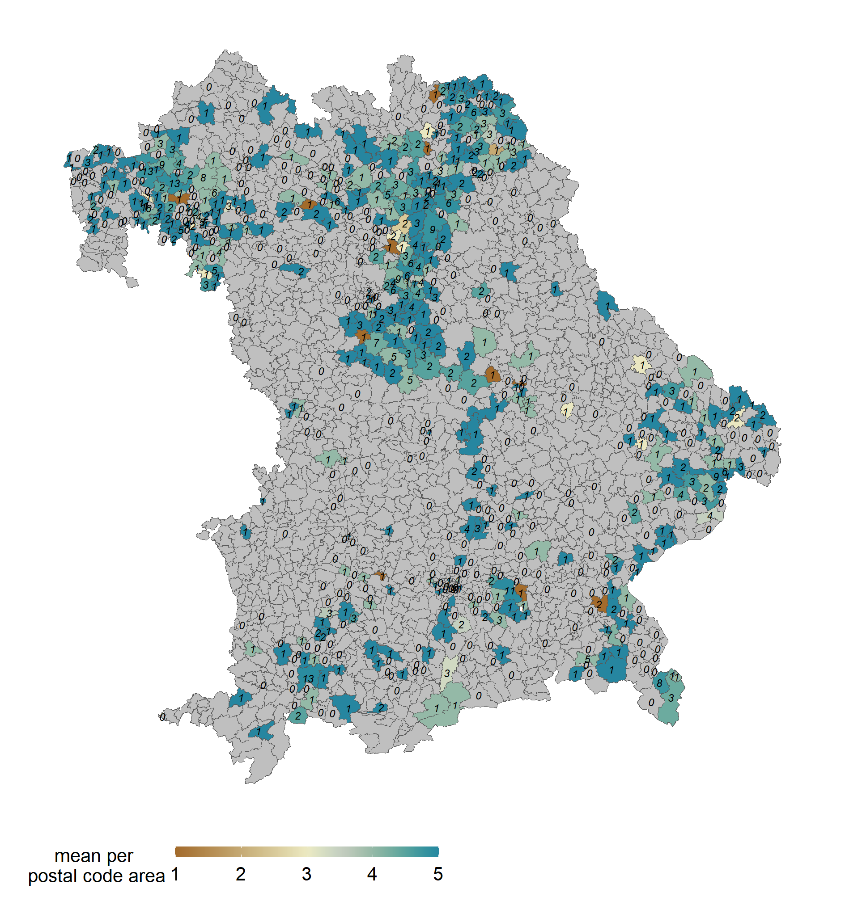
**

*Figure S20. Perceived importance of protection of groundwater quality. The small number within each postal code polygon represents the number of respondents from this area.*

**
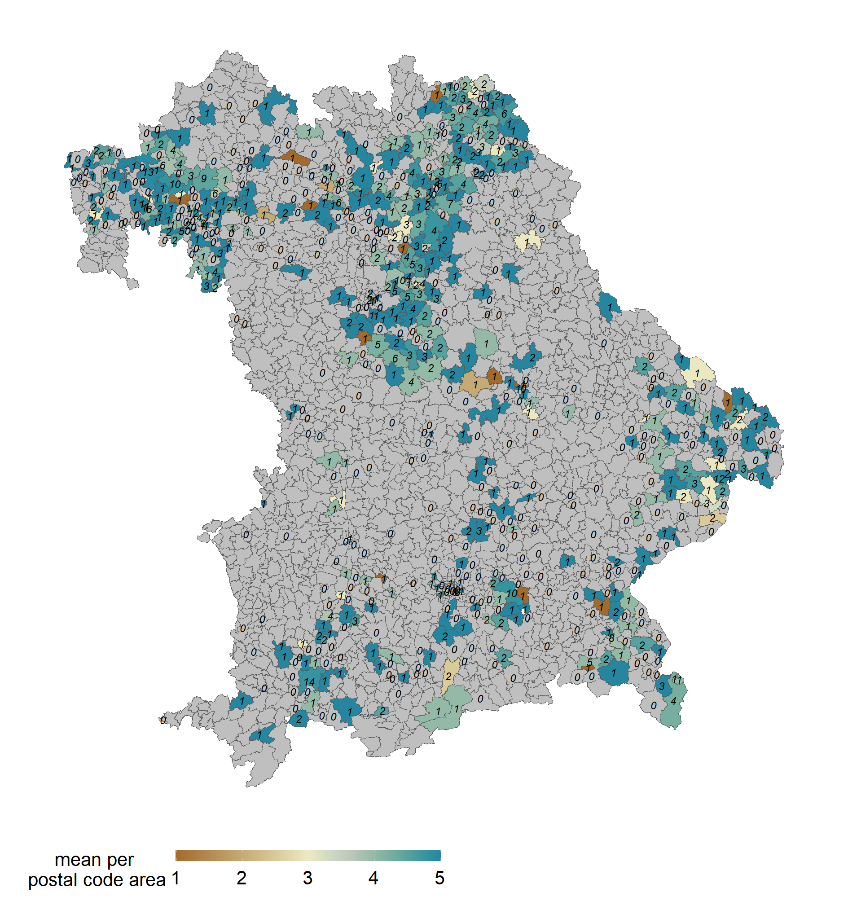
**

*Figure S21. Perceived importance of groundwater formation. The small number within each postal code polygon represents the number of respondents from this area.*

**
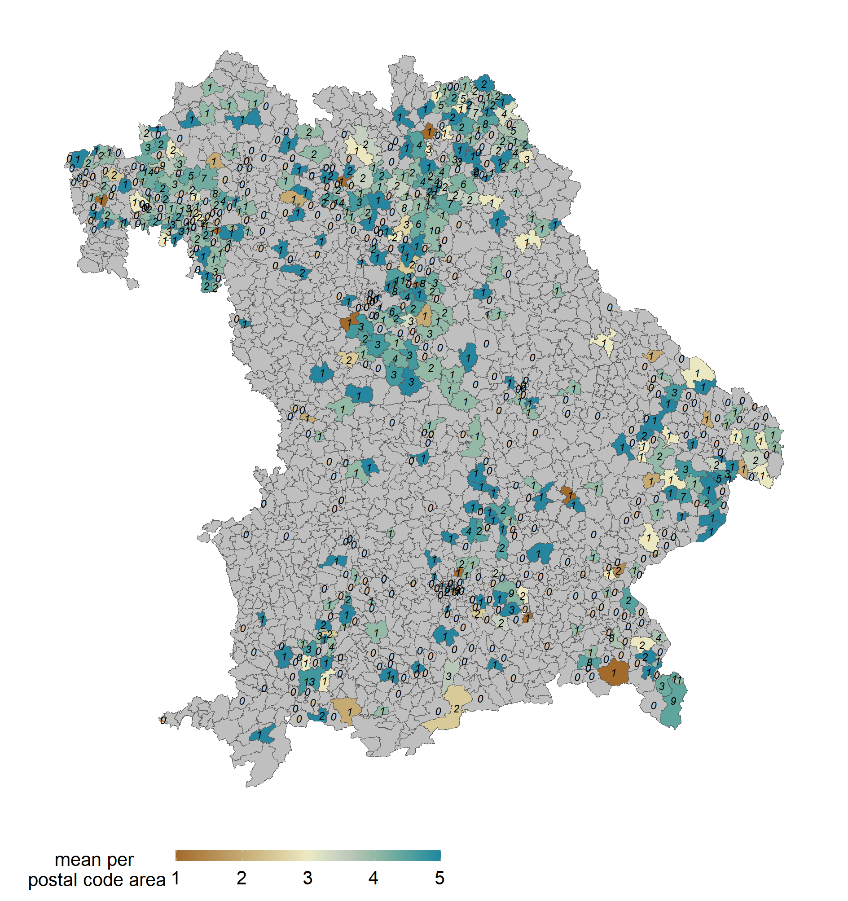
**

*Figure S22. Perceived importance of flood control. The small number within each postal code polygon represents the number of respondents from this area.*

**
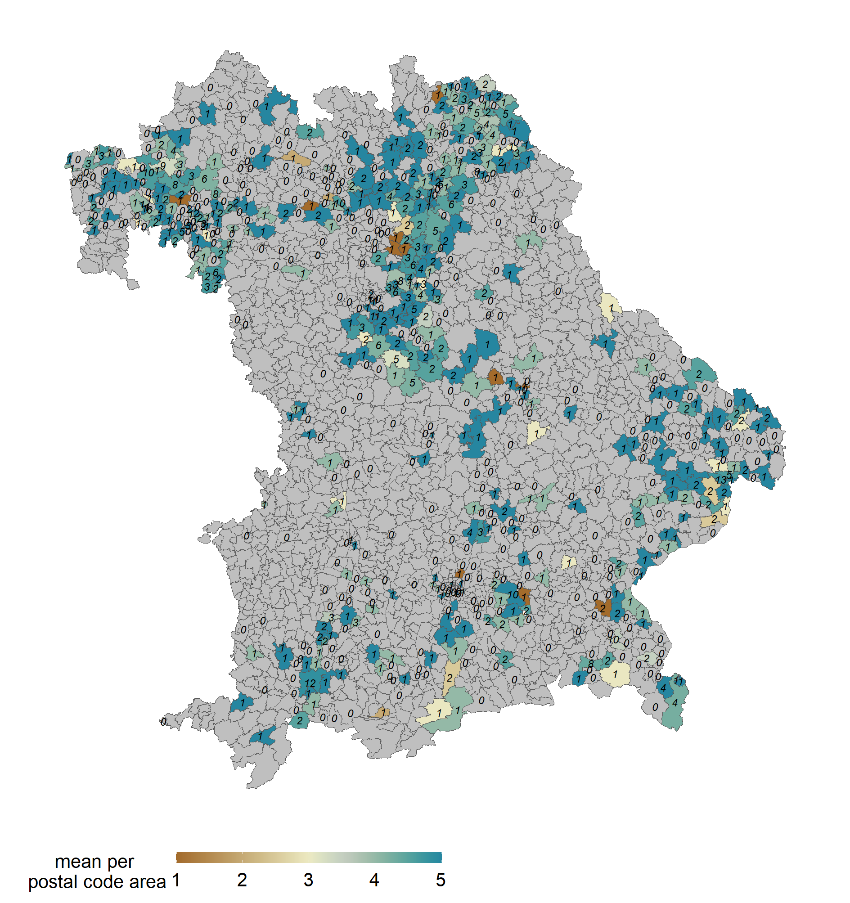
**

*Figure S23. Perceived importance of air purification. The small number within each postal code polygon represents the number of respondents from this area.*

**
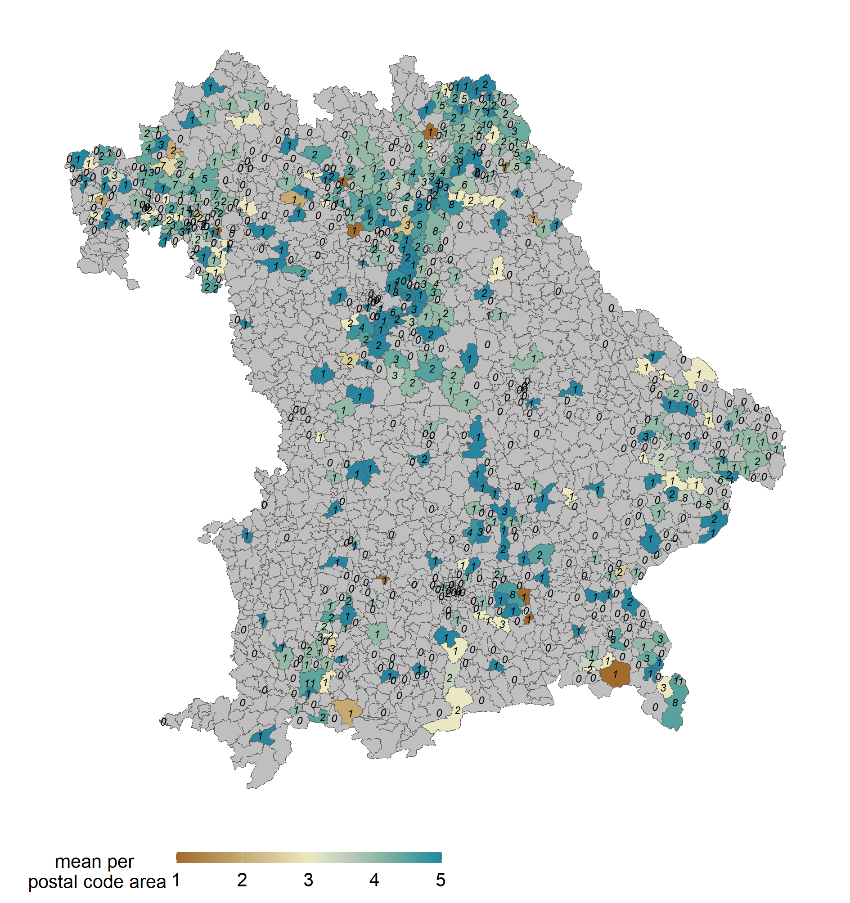
**

*Figure S24. Perceived importance of local climate regulation. The small number within each postal code polygon represents the number of respondents from this area.*

**
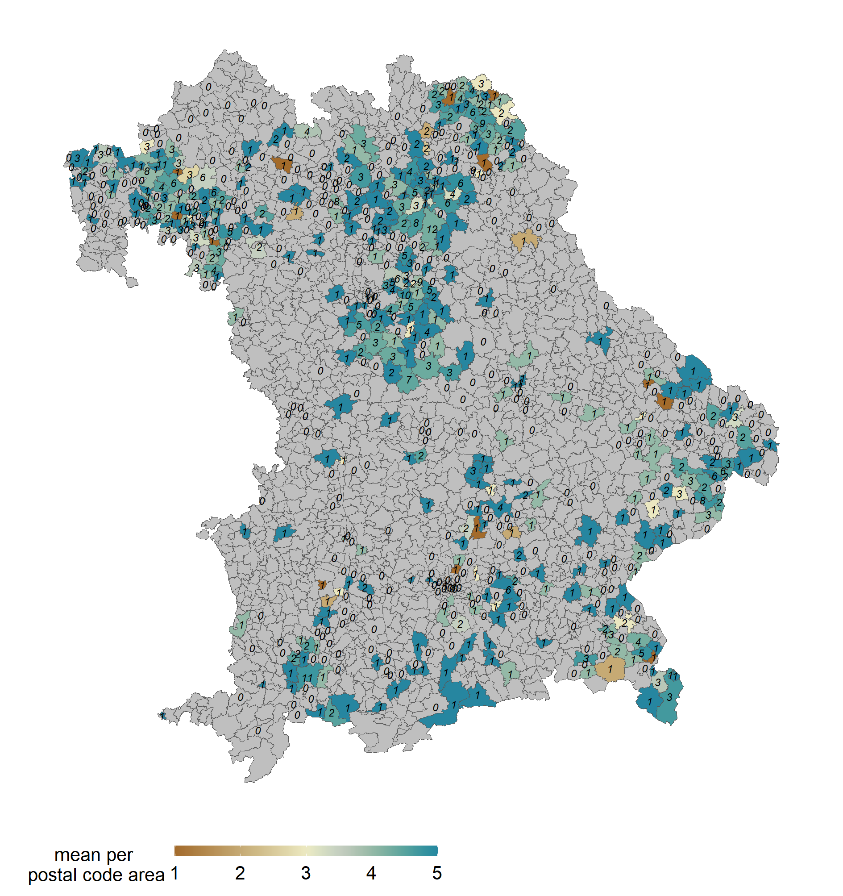
**

*Figure S25. Perceived importance of global climate regulation. The small number within each postal code polygon represents the number of respondents from this area.*

**
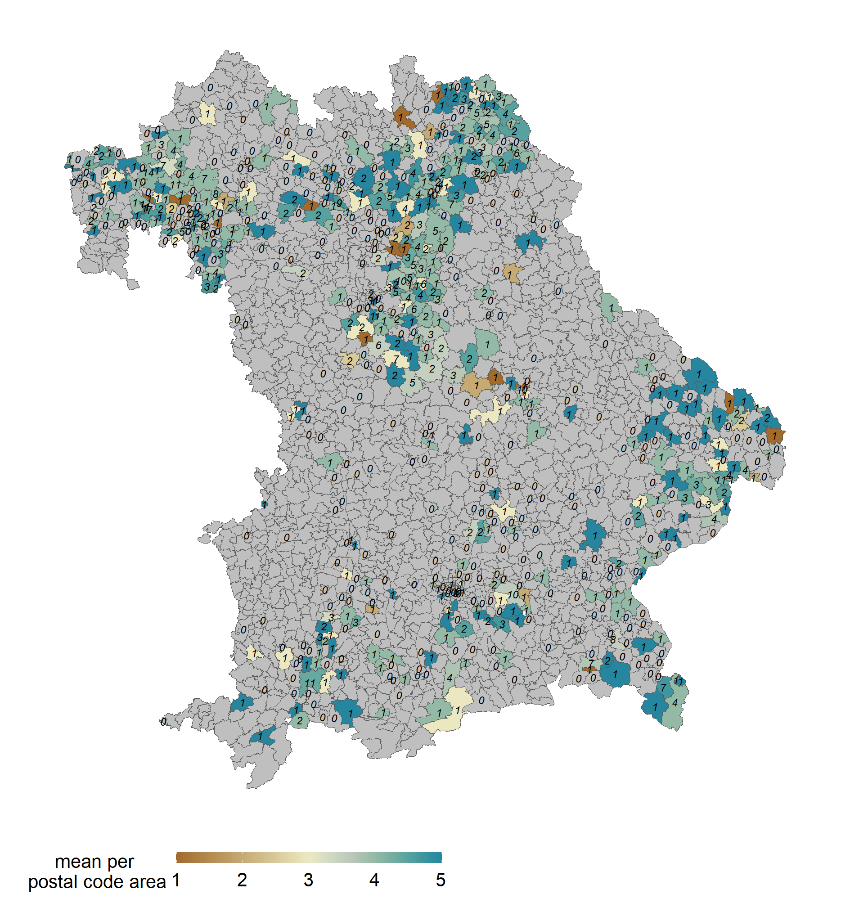
**

*Figure S26. Perceived importance of sense of place. The small number within each postal code polygon represents the number of respondents from this area.*

**
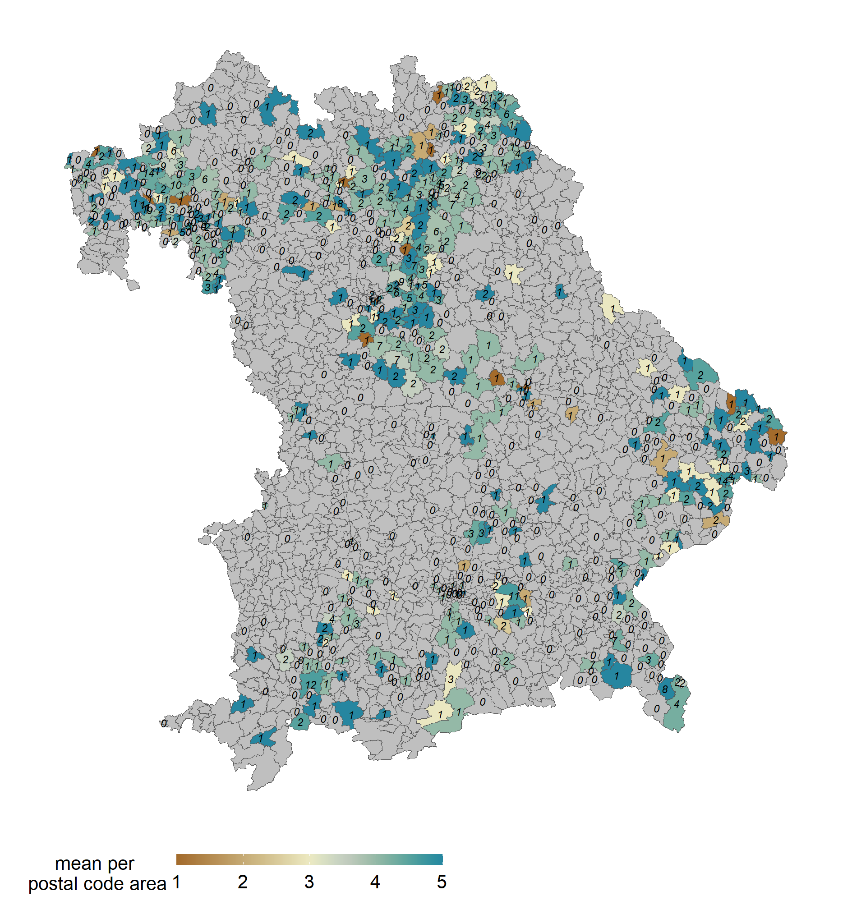
**

*Figure S27. Perceived importance of recreation in open landscapes. The small number within each postal code polygon represents the number of respondents from this area.*

**
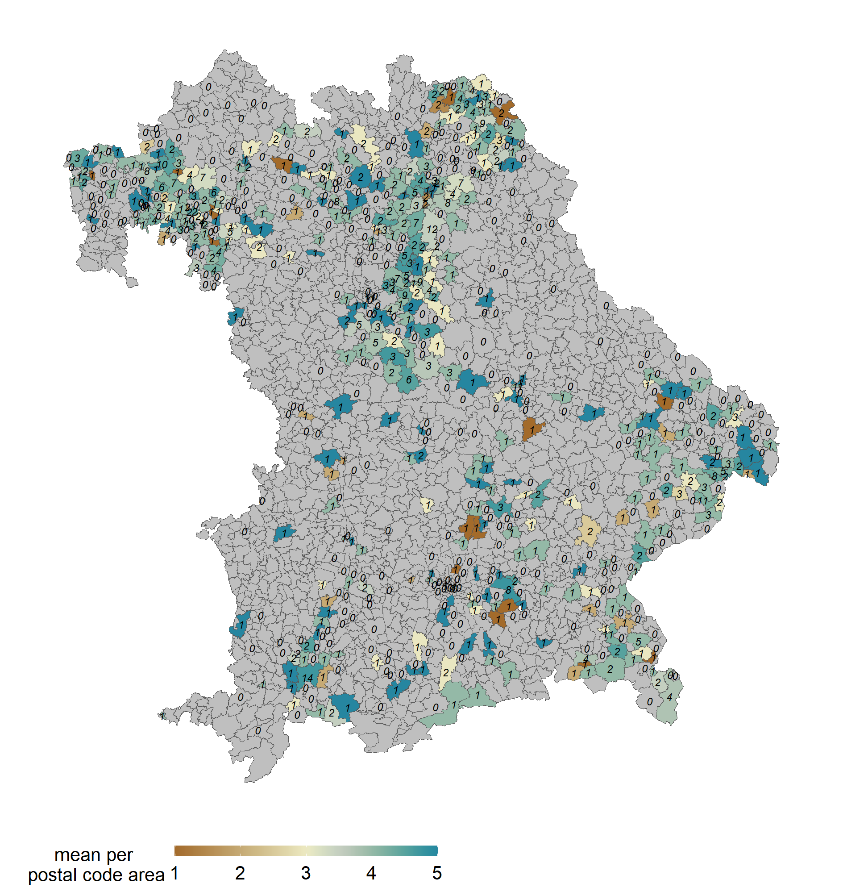
**

*Figure S28. Perceived importance of recreation in urban areas. The small number within each postal code polygon represents the number of respondents from this area.*

**
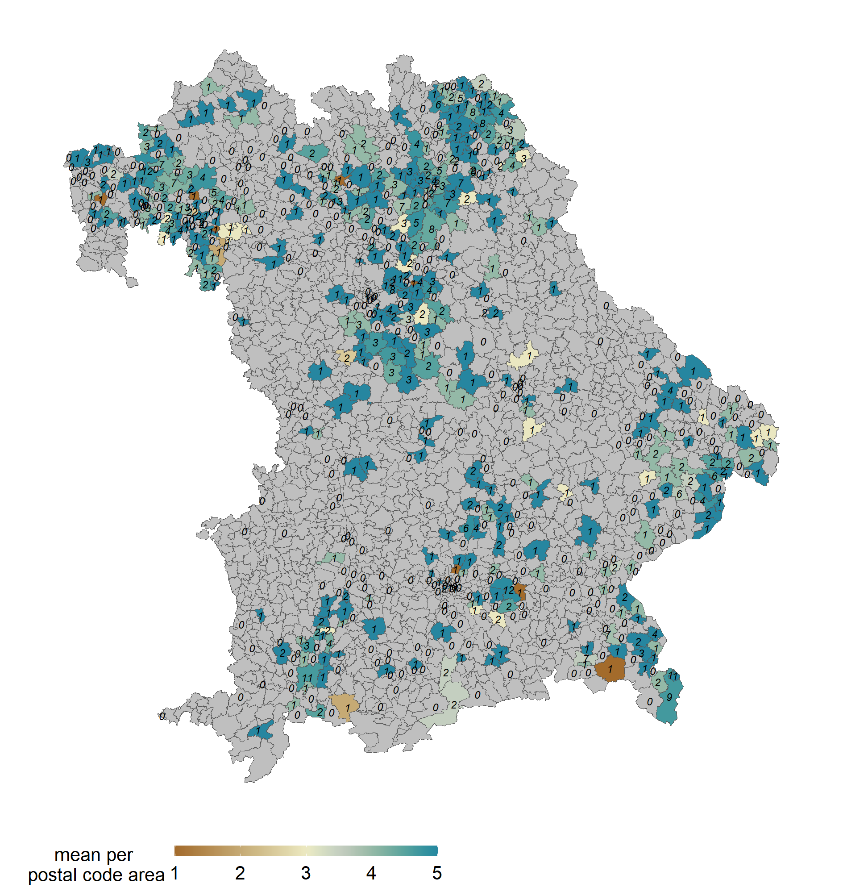
**

*Figure S29. Perceived importance of habitat. The small number within each postal code polygon represents the number of respondents from this area.*

**
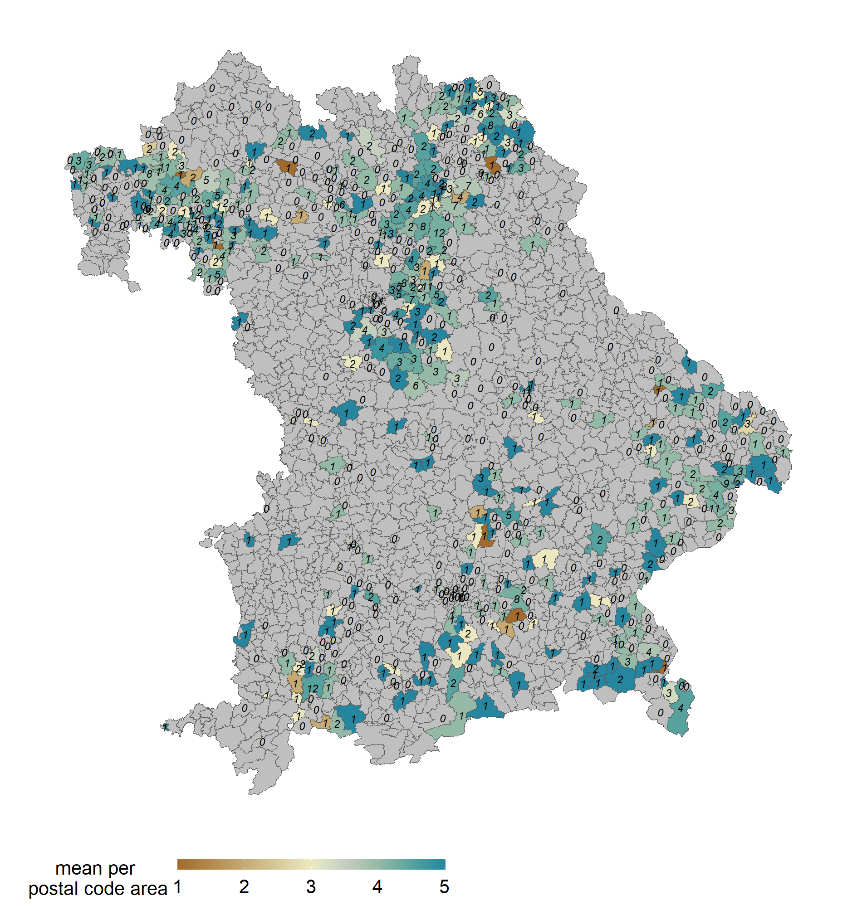
**

*Figure S30. Perceived importance of learning and inspiration. The small number within each postal code polygon represents the number of respondents from this area.*

Distribution of residuals per significant regression model across Bavaria


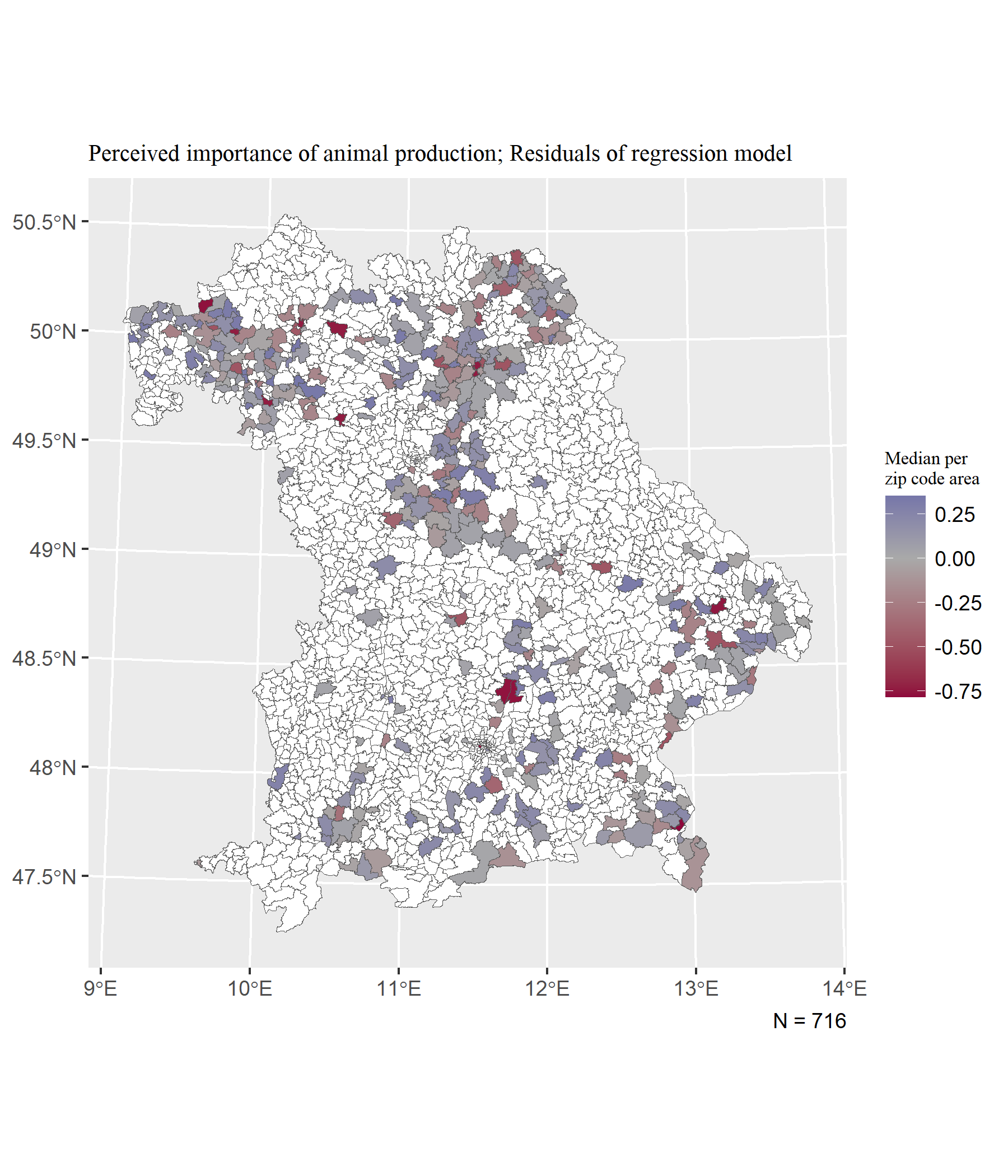


*Figure S31. Residuals of regression model: Perceived importance of animal production in the open field.*


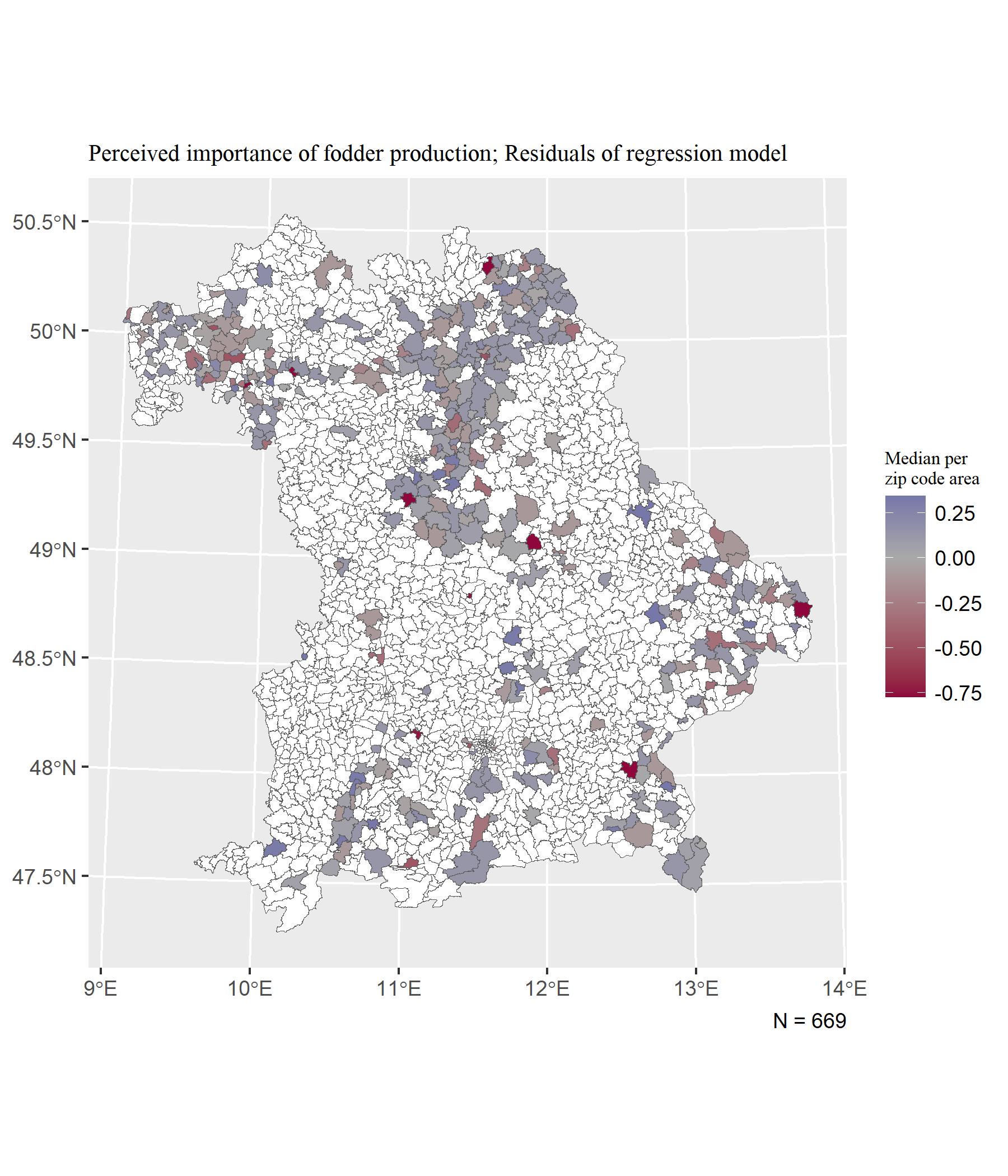


*Figure S32. Residuals of regression model: Perceived importance of fodder production.*


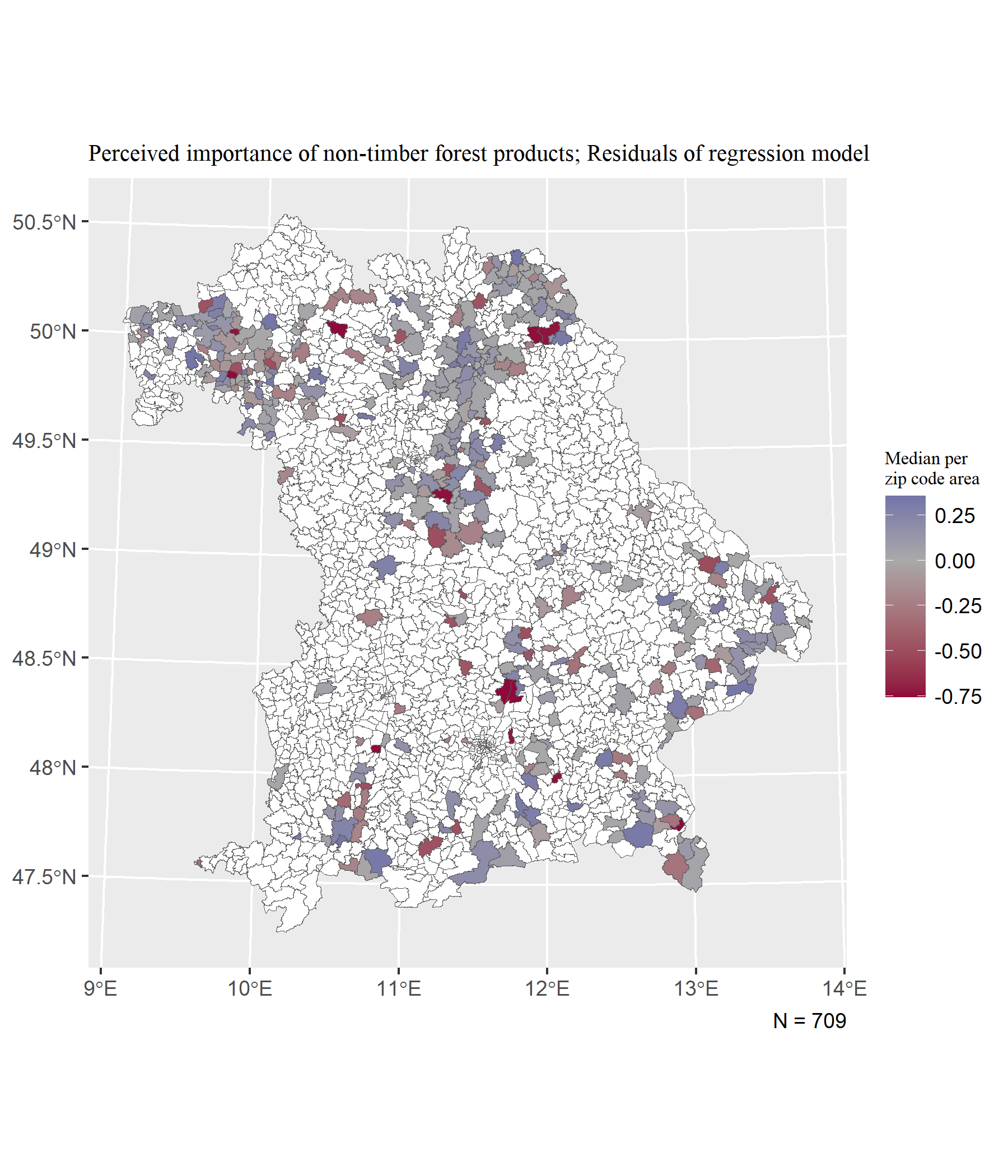


*Figure S33. Residuals of regression model: Perceived importance of non-timber forest products.*


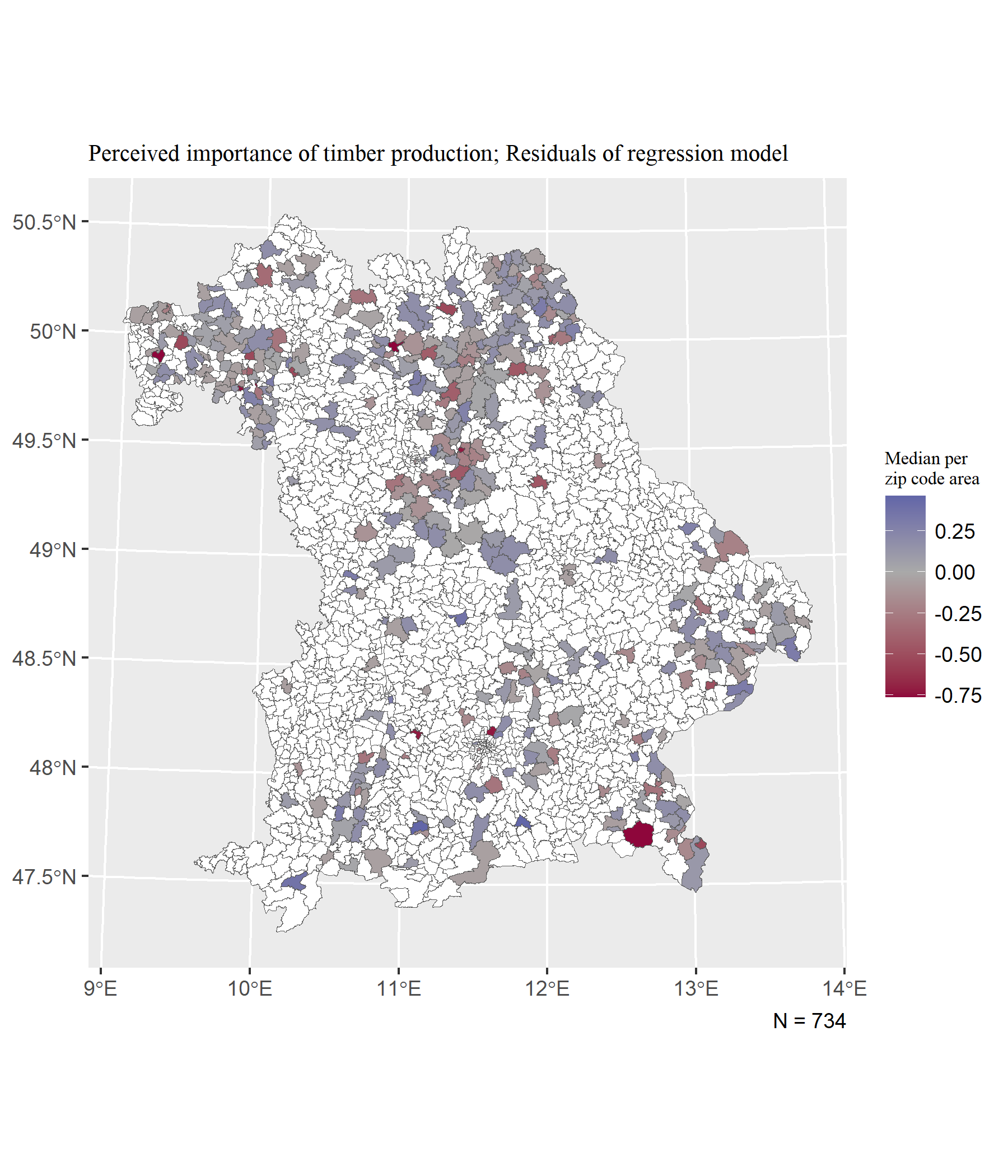


*Figure S34. Residuals of regression model: Perceived importance of timber production.*


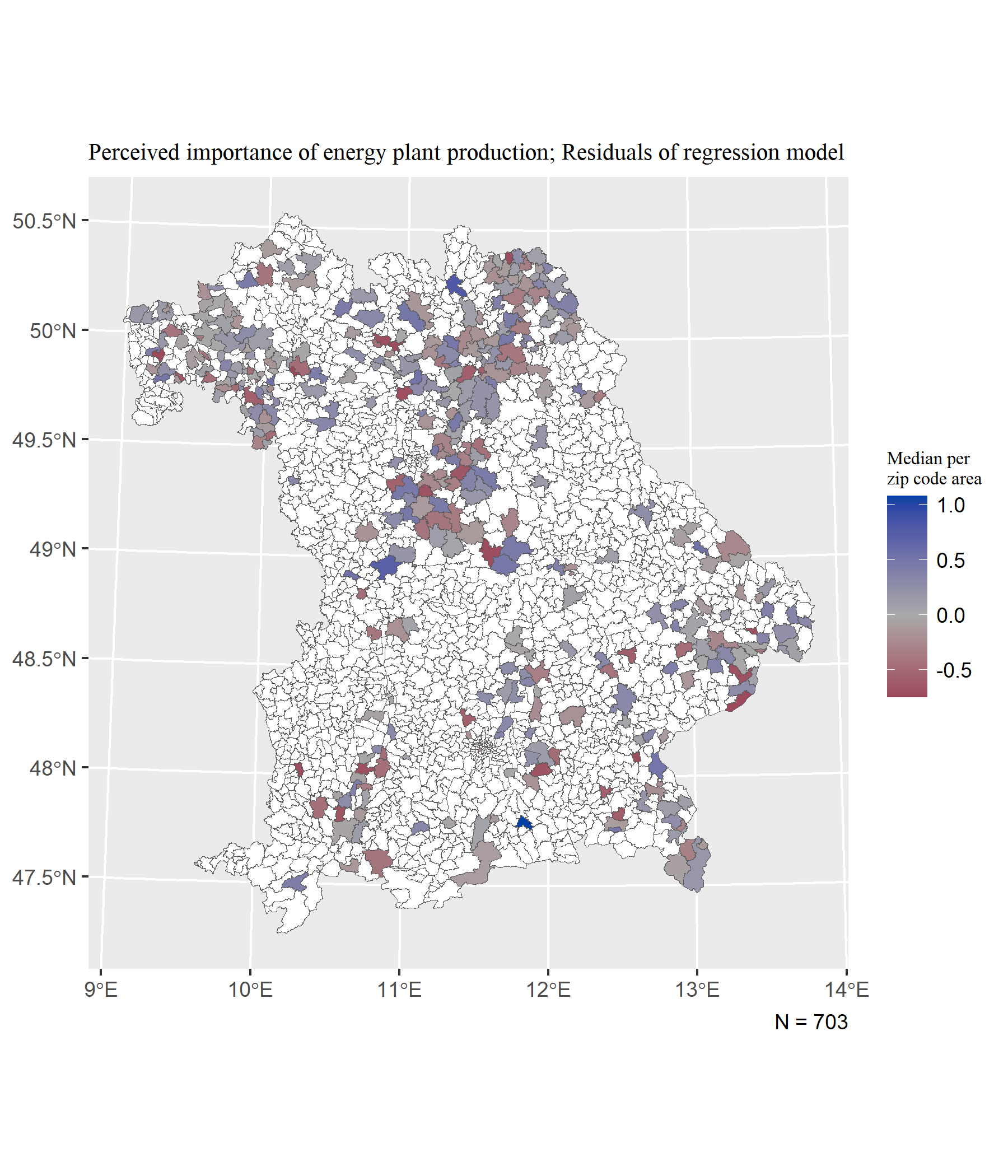


*Figure S35. Residuals of regression model: Perceived importance of energy plant production.*


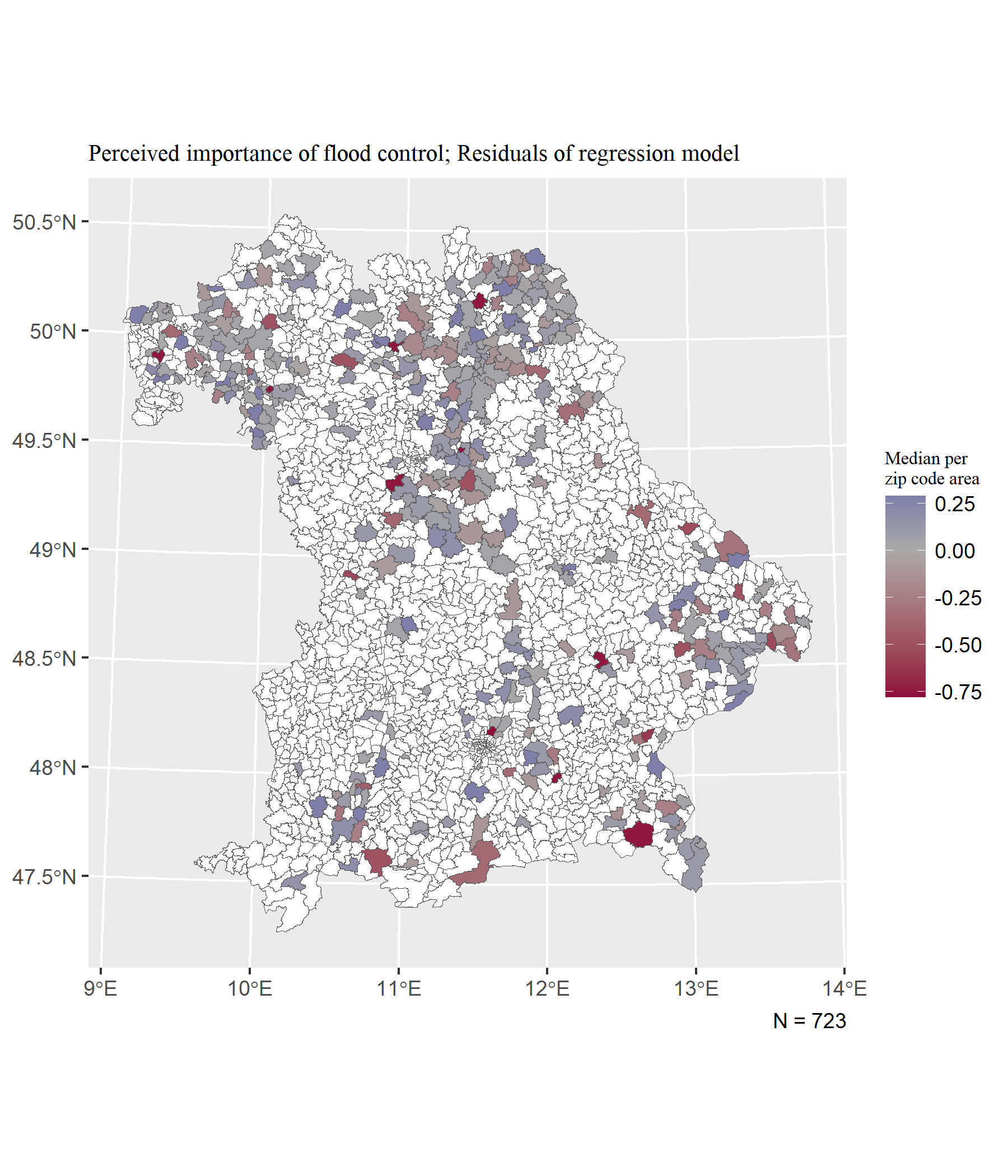


*Figure S36. Residuals of regression model: Perceived importance of flood control.*


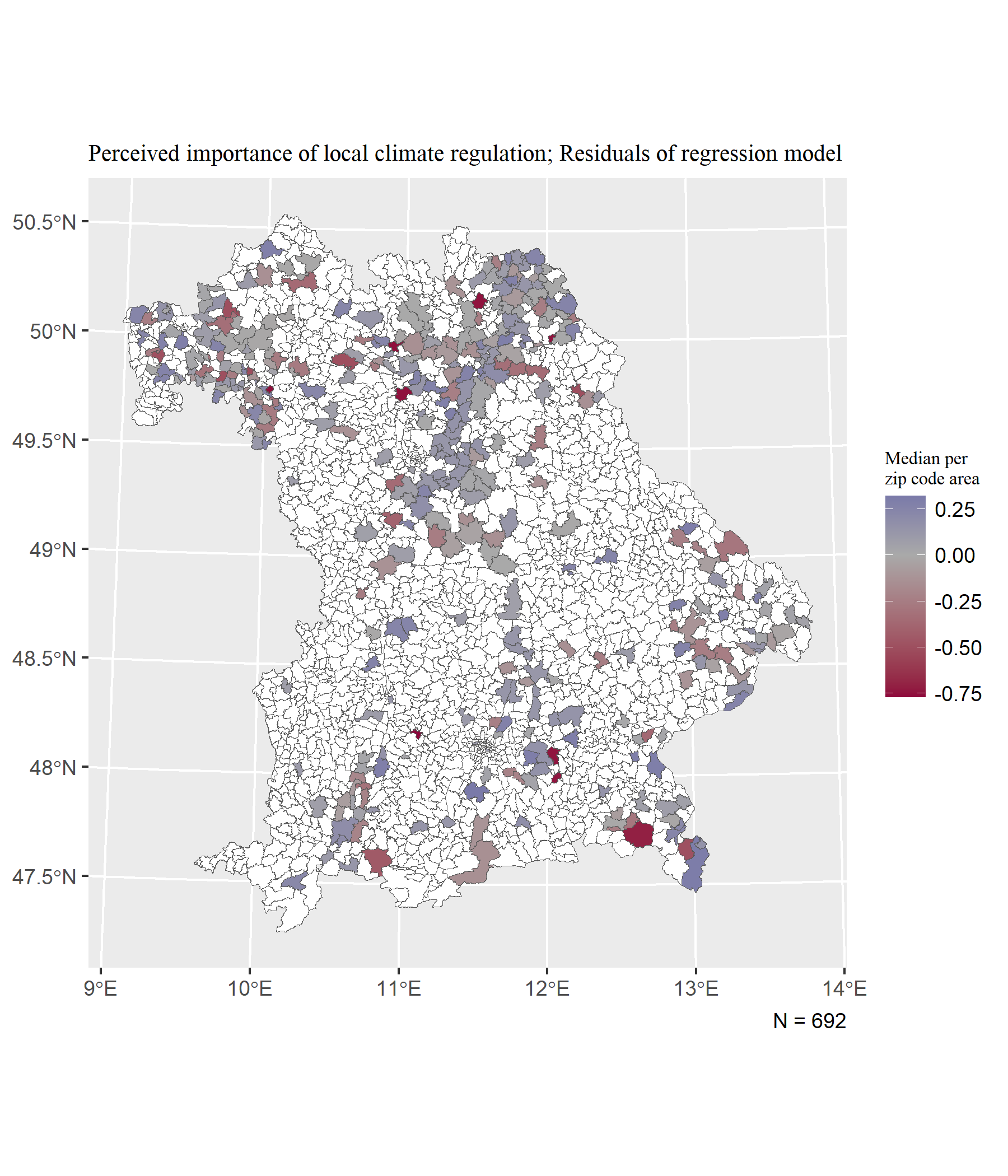


*Figure S37. Residuals of regression model: Perceived importance of local climate regulation.*


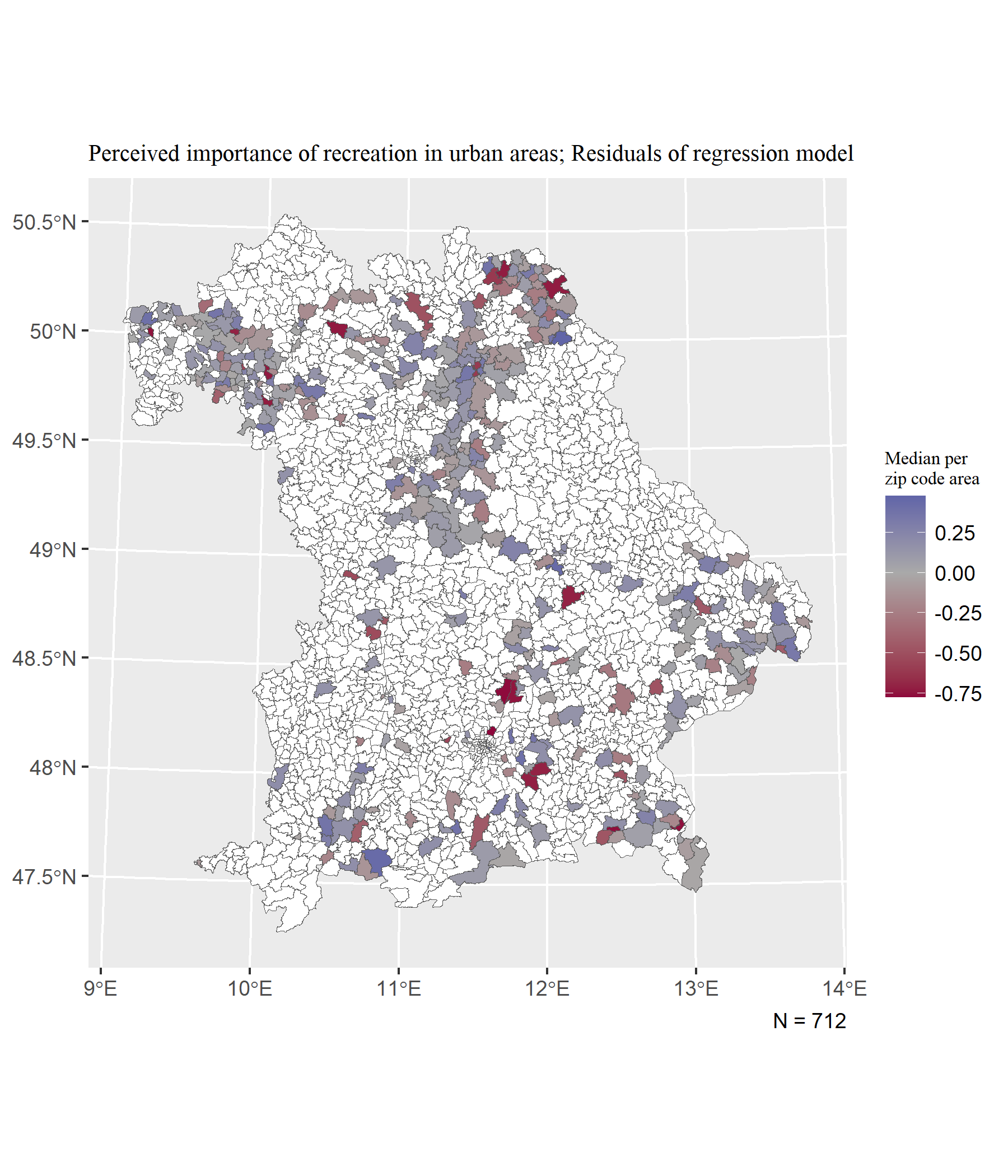


*Figure S38. Residuals of regression model: Perceived importance of recreation in urban areas.*


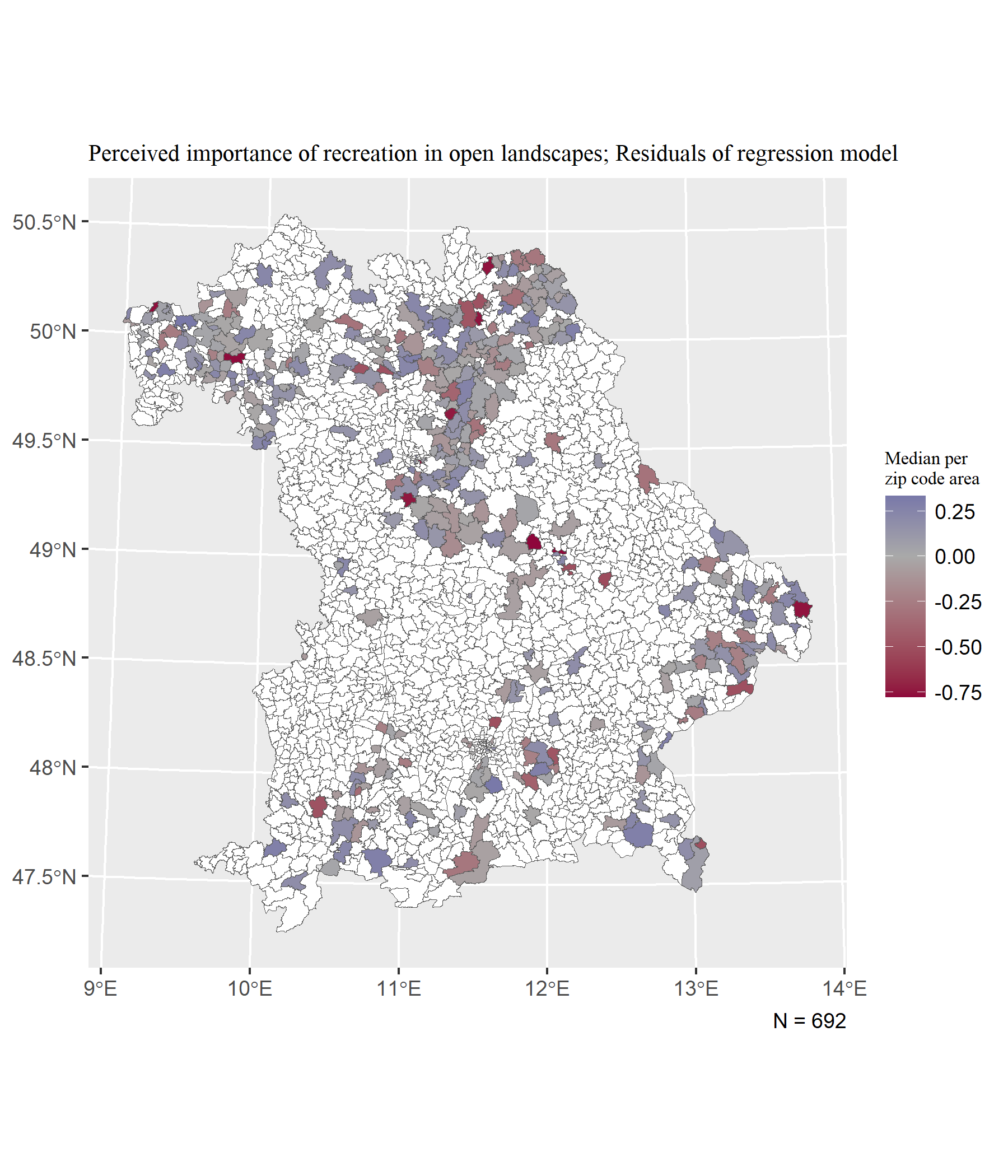


*Figure S39. Residuals of regression model: Perceived importance recreation in open landscapes.*


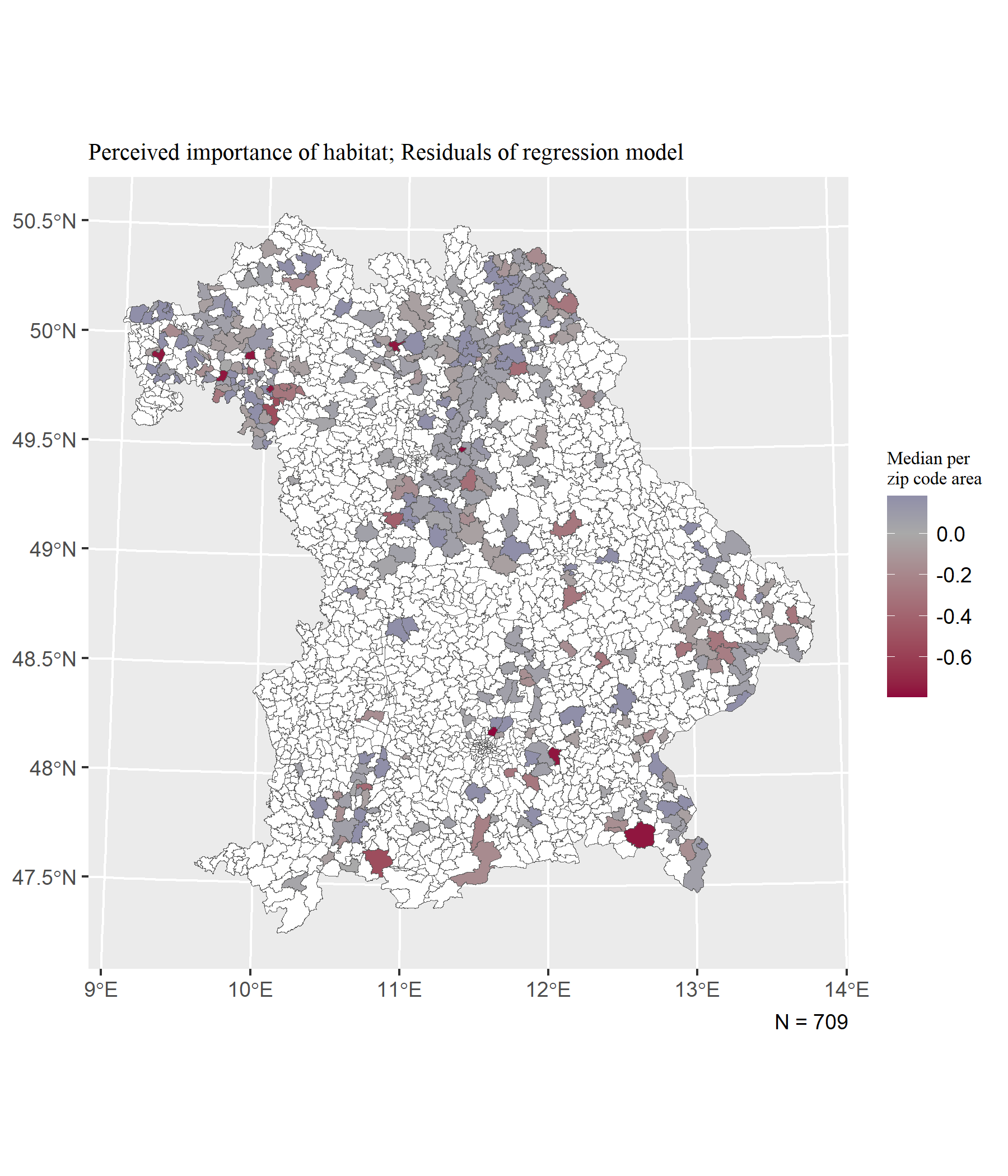


*Figure S40. Residuals of regression model: Perceived importance of habitat.*


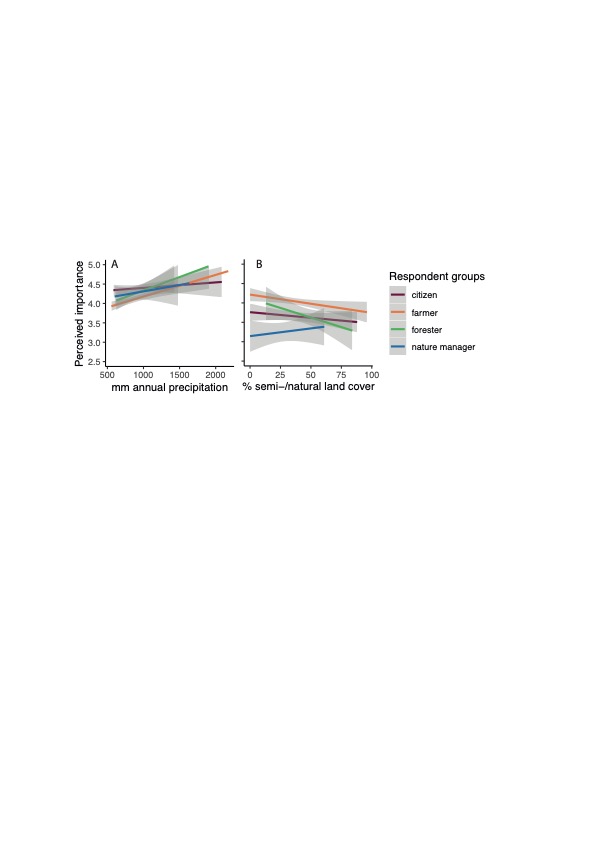


Figure S41. Regression lines from the GLMs for significant relations. A) Perceived importance of energy plant production in dependence of natural adjacent land cover. B) Perceived importance of animal production in the open field increasing with annual mean precipitation.
